# Troxerutin suppresses the stemness of hepatocellular carcinoma via the Syk/FOXO3 feedback loop

**DOI:** 10.1007/s10565-026-10194-z

**Published:** 2026-05-08

**Authors:** Shan Liu, Yuan Lin, Huan Xia, Hanhan Li, Yan Dou, Jiamin Luo, Tiantian Yang, Qi Zeng, Wei Guo, Hanrui Chen

**Affiliations:** 1 Science and Technology Innovation Center, Guangzhou University of Chinese Medicine, Guangzhou, 510006 Guangdong China; 2 Department of Pathology, The First Affiliated Hospital of Sun Yat Sen University, Guangzhou, 510080 Guangdong China; 3 Institute of Hydrobiology, Jinan University, Guangzhou, 510632 Guangdong China; 4 Geriatrics Research Institute, Sichuan Provincial People’s Hospital, University of Electronic Science and Technology of China, Chengdu, 610072 Sichuan China; 5 Department of Oncology, The First Affiliated Hospital of Guangzhou University of Chinese Medicine, Guangzhou, 510006 Guangdong China; 6 The Second Clinical Medical College, Guangzhou University of Chinese Medicine, Guangzhou, 510006 Guangdong China; 7 Shunde Hospital of Guangzhou University of Chinese Medicine, Guangzhou University of Chinese Medicine, Guangzhou, 528300 Guangdong China

**Keywords:** Troxerutin, Hepatocellular carcinoma Stemness, FOXO3/Syk feedback loop

## Abstract

Hepatocellular carcinoma (HCC) remains one of the deadliest malignancies worldwide due to its high rates of recurrence and metastasis. This underscores the urgent need to develop innovative drugs and treatment strategies to improve patient outcomes. This study examined the molecular pathways underlying troxerutin's therapeutic potential in HCC, providing information that could aid in the development of novel therapeutic strategies. Cell proliferation and apoptosis were assessed using CCK-8, EdU assays, and flow cytometry. Protein expression and mRNA alterations were analyzed by Western blotting and PCR, respectively. Cell migration and invasion were evaluated using Transwell assays, while tumor cell stemness was examined through sphere formation assays. Troxerutin's functional role was studied using in vivo models. Proteomic profiling of troxerutin-treated cells was performed using iTRAQ combined with LC–MS/MS to identify differentially expressed proteins (DEPs). Furthermore, luciferase reporter and ChIP assays were conducted to elucidate the underlying regulatory mechanisms. These findings demonstrate that troxerutin markedly inhibits HCC malignancy and stemness in both in vitro and in vivo models. Bioinformatics analysis indicated that the FOXO and SYK signaling pathways were predominantly enriched among the differentially expressed proteins. Mechanistically, we show that Syk dephosphorylation activates FOXO3 and facilitates its translocation into the nucleus. Moreover, we confirm that FOXO3 directly associates with the Syk promoter, thereby initiating its transcription. The present study reveals that troxerutin attenuates HCC progression by targeting the FOXO3/Syk feedback loop, inhibiting the stem-like properties of HCC cells.

**Supplementary Information:**

The online version contains supplementary material available at 10.1007/s10565-026-10194-z.

## Introduction

Hepatocellular carcinoma (HCC) ranks among the most prevalent and lethal cancers worldwide, yet conventional treatments have limited success in reducing metastasis and recurrence (Gilles et al. 2022). Recent advances in systemic therapies, such as immune checkpoint inhibitors and combination regimens, have improved treatment options for advanced HCC (Sensi et al. 2024). However, responses are variable, and challenges like immune resistance and the complex tumor microenvironment remain. Resistance and relapse in HCC are primarily attributed to abnormal cell differentiation and the presence of cancer stem cells (CSCs) (Stouras et al. 2023). CSCs are a small subset of tumor cells with self-renewal and differentiation potential. They also have strong tumorigenicity and are considered key drivers of tumor initiation and progression (Darie et al. 2026). In HCC, CSCs exhibit markedly higher spheroid-forming capacity and greater resistance to sorafenib compared to non-CSCs (Yousef et al. 2025). In addition to CSCs, dysregulation of specific molecular regulators, such as PRDX6, can drive HCC progression through multiple signaling pathways. This underscores the complex molecular landscape of HCC (Mu et al. 2024). Clinical analyses have also identified Vav1 as a marker associated with tumor differentiation, stage, recurrence, and overall survival, underlining the importance of characterizing prognostic molecules in HCC (Ye et al. 2024). As a result, therapies targeting CSCs offer a promising strategy for improving HCC treatment outcomes. Natural compounds, including Icaritin, can target specific molecular pathways in HCC, providing a rationale to investigate troxerutin as a potential modulator of tumor progression (Zhou et al. 2024b).

Troxerutin, a naturally occurring flavonoid, possesses a broad range of pharmacological activities, notably strong antioxidant and anti-inflammatory effects demonstrated in both in vitro and in vivo studies (Demir et al. 2025; Mohiuddin et al. 2025). Studies have shown its cytoprotective effects through free radical scavenging capabilities, as well as its ability to attenuate inflammatory responses by reducing inflammatory cell infiltration and suppressing pro-inflammatory cytokines and related proteins (Chen et al. 2025b; Demir et al. 2025; Gamal et al. 2025). These properties underlie its clinical utility in managing chronic venous insufficiency and other vascular disorders. Emerging evidence has expanded the therapeutic potential of troxerutin, revealing anti-diabetic, anti-tumor, and neuroprotective effects (Gamal et al. 2025; Mohiuddin et al. 2025; Sowunmi et al. 2024; Wu et al. 2025). Importantly, recent studies have documented its efficacy in Alzheimer's disease, colorectal cancer, and osteosarcoma models (Chen et al. 2025a; Zamanian et al. 2021). However, despite these promising applications, the therapeutic mechanisms of troxerutin in HCC remain poorly characterized.

Overexpression of spleen tyrosine kinase (Syk) has been linked to the progression of multiple cancer types (Fueyo et al. 2018). Syk is a cytoplasmic non-receptor protein tyrosine kinase that plays a key role in immune cell signaling. It is predominantly expressed in hematopoietic-derived cells, including mast cells, B cells, neutrophils, and macrophages (Krisenko and Geahlen 2015). In cancer, elevated Syk expression acts as a tumor promoter, influencing immune regulation, drug responsiveness, and key processes such as invasion, migration, apoptosis, and tumor cell proliferation (Iqbal et al. 2025). However, the role of troxerutin in modulating Syk during malignant progression is poorly studied and warrants further investigation.

This study investigated the effects of troxerutin in HCC, demonstrating through both in vitro and in vivo experiments that troxerutin markedly suppresses malignant progression. Western blotting showed that Syk dephosphorylation triggers FOXO3 activation and promotes its nuclear translocation. In this study, we focused on the Syk/FOXO3 axis and did not examine possible interactions with other pathways such as Wnt or Notch. Using bioinformatics analysis, dual-luciferase, and ChIP assays, we confirmed that FOXO3 directly binds to the Syk promoter to initiate its transcription. These results indicate that troxerutin suppresses HCC progression by modulating the FOXO3/Syk signaling axis. The findings of this study highlight the therapeutic potential of troxerutin as a strategy to target Syk and inhibit HCC malignancy, offering a promising approach for HCC treatment.

## Materials and methods

### Cell culture and transfection

The highly metastatic human HCC cell lines HCC-LM3 (IM-H037, IMMOCELL, Xiamen, China) and MHCC97H (IM-H045, IMMOCELL, Xiamen, China) were obtained from a commercial supplier and cultured in DMEM with 10% FBS and 1% penicillin–streptomycin. The cells were cultured in a humidified incubator at 37 °C with 5% CO₂ under normal conditions. Cultures were passaged at a 1:3 ratio every six days, with medium changes performed every other day. Cells used in this study were maintained within a limited passage range after resuscitation, and all the cell lines were authenticated using Short Tandem Repeats (STR) typing test by supplier.

To knock down Syk and FOXO3, shRNA and control constructs were purchased from Genepharma (Shanghai, China), whereas Syk and FOXO3 were overexpressed by preparing a pcDNA3.0 vector harboring their coding sequence. Following the manufacturer's instructions, Lipofectamine 2000 (Invitrogen, CA, USA) was used to transfect cells at 80–90% confluency. After 6 h of transfection, the medium was changed, and 24 to 48 h later, cells were extracted for use in further processes.

### RNA isolation and quantitative real-time PCR (qRT‑PCR) assay

TRIzol reagent (Invitrogen, CA, USA) was used to extract total RNA, and the PrimeScript RT reagent kit (Takara, Japan) was used for constructing cDNA. GAPDH served as the internal control for the quantitative PCR, which was carried out using an ABI 7900HT Fast Real-Time PCR System (Applied Biosystems, USA) using SYBR Green SuperMix (Roche, Switzerland). The cycling conditions were as follows: 40 cycles of 94 °C for 30 s, 55 °C for 30 s, and 72 °C for 90 s. Relative gene expression was calculated using the 2^⁻ΔΔCt^ method. These analyses used the following primers: OCT4F: 5ˊ- CCTTCGCAAGCCCTCATTTC −3ˊ, R: 5ˊ- TAGCCAGGTCCGAGGATCAA −3ˊ; SOX2F: 5ˊ- CATGAAGGAGCACCCGGATT −3ˊ, R: 5ˊ- TAACTGTCCATGCGCTGGTT −3ˊ; NANOGF: 5ˊ- GTCTCGTATTTGCTGCATCGT-3ˊ, R: 5ˊ-TTCCTTCTCCACCCCAACCA-3ˊ; KLF4 5ˊ- TTTGTGGGCCTGAAGAAAACT −3ˊ, R: 5ˊ- AGGGCTGTCCTGAATAAGCAG −3ˊ; GAPDH F: 5'-GACAGTCAGCCGCATCTTCT-3', and GAPDH R: 5'-GCGCCCAATACGACCAAATC-3'.

### CCK-8 assay

Cell proliferation was evaluated using a CCK‑8 kit (Yeasen, China). Cells were seeded in 96‑well plates at 5 × 10^3^ cells per well in 100 µL of complete medium and incubated for 24 h to allow adherence. Afterward, cells were treated with serial dilutions of the test compound (ranging from 0.1 µM to 100 µM, in triplicate) and incubated for 48 h. Control wells contained cells with medium only (untreated control) and medium without cells (blank control). After treatment, each well received 10 µL of CCK-8 reagent (Dojindo, Japan), which was then incubated for 1–4 h at 37 °C. A microplate reader (BioTek, USA) was used to detect absorbance at 450 nm, and the following formula was used to determine cell viability:

$$\mathrm{Viability}\left(\mathrm{\%}\right)=\frac{{\mathrm{OD}}_{\mathrm{drug}-\mathrm{treated}}-{\mathrm{OD}}_{\mathrm{blank}}}{{\mathrm{OD}}_{\mathrm{control}}-{\mathrm{OD}}^{\mathrm{blank}}}$$

The half‑maximal inhibitory concentration (IC₅₀) was calculated by fitting dose–response curves to a four‑parameter logistic (4PL) model using GraphPad Prism 9.0 (GraphPad Software, USA).

### EdU (5-ethynyl-20-deoxyuridine) assay

Cell proliferation was assessed using an EdU assay kit (Ribobio, Guangzhou, China). Cells were seeded in confocal plates at 1 × 10^5^ cells/well, treated with 50 μM EdU, and incubated at 37 °C for 2 h. Cells were fixed for 30 min with 4% formaldehyde, then permeabilized for 20 min with 0.1% Triton X-100, stained with EdU solution, and counterstained with Hoechst. A fluorescent microscope was then used to take fluorescence pictures.

### Flow cytometry analysis

Following transfection, HCC cells were grown in 6‑well plates to 90% confluence, harvested, and stained with 10 μL of Annexin V‑FITC/propidium iodide (PI) from an apoptosis detection kit (Lianke Biotech Co. Ltd., Hangzhou, China) for 15 min at room temperature in the dark. Apoptosis was then analyzed using a flow cytometer (FACSCalibur, BD Biosciences, USA).

### Transwell assays

Cell migration and invasion were assessed using Transwell chambers (Corning) with or without Matrigel coating. In the upper chamber, cells were planted in serum-free media, and in the bottom chamber, they were allowed to migrate toward medium containing 10% FBS. The migrated cells underwent staining, imaging, and measurement.

### Sphere formation assay

For sphere formation assays, cells were harvested and seeded at a density of 1,000 cells per well in nonadherent six‑well plates (Costar; Corning) containing serum‑free DMEM. After 3 days, an equal volume of fresh medium was added, and cultures were maintained for an additional 7 days to allow sphere development. Formed spheres were then visualized and imaged using a phase‑contrast microscope.

### Western blot assay

RIPA lysis buffer enhanced with a protease inhibitor cocktail was used to extract the proteins. Nuclear extracts from HCC‑LM3 and MHCC‑97H cells were prepared using a Nuclear and Cytoplasmic Protein Extraction Kit (Beyotime, Wuhan, China). Samples were separated on 8–12% SDS-PAGE gels and then transferred onto 0.45 μm PVDF membranes (Millipore, USA) after protein concentrations were assessed using a BCA assay. Membranes were blocked with skimmed milk, incubated with primary antibodies overnight at 4 °C, and then probed with HRP‑conjugated secondary antibodies (Proteintech). Enhanced chemiluminescence was used to visualize the protein bands, and ImageJ software was used to quantify band intensities. Data were normalized to GAPDH for cytoplasmic and total proteins, or to Histone H3 for nuclear proteins. Antibodies used included: NANOG (1:30,000, Proteintech Cat# 14,295–1-AP, RRID:AB_1607719), OCT‑4 (1:3000, Proteintech Cat# 11,263–1-AP, RRID:AB_2167545), SOX2 (1:1000, Proteintech Cat# 11,064–1-AP, RRID:AB_2195801), KLF4 (1:5000, Proteintech Cat# 11,880–1-AP, RRID:AB_10640807), c‑MYC (1:8000, Proteintech Cat# 10,828–1-AP, RRID:AB_2148585), p‑Syk (1:20,000, Proteintech Cat# 83,331–1-RR, RRID:AB_3670995), Syk (1:6000, Proteintech Cat# 66,721–1-Ig, RRID:AB_2882072), p‑FOXO3 (1:1000, Proteintech Cat# 28,755–1-AP, RRID:AB_2881210), FOXO3 (1:5000, Proteintech Cat# 10,849–1-AP, RRID:AB_2247214), Histone H3 (1:10,000, Proteintech Cat# 17,168–1-AP, RRID:AB_2716755), and GAPDH (1:250,000, Proteintech Cat# 60,004–1-Ig, RRID:AB_2107436).

### Prediction of troxerutin targets for Syk

Syk were selected as the corresponding ligands for the hub genes, and the 2D structures of these ligand molecules were downloaded from the PubChem database (https://Pubchem.ncbi.nlm. nih.gov/). The RCSB PDB database (https://www.rcsb.or g/) was used to retrieve and download the protein structure files of the target genes (Syk; ID: 4puz).AutoDock software (Autoock_vina_1_2_3) was used to perform molecular docking of the target genes and ligands.

### Dual luciferase assay

FOXO3 binding sites within the human Syk promoter region were predicted using the JASPAR database. Wild‑type and mutant Syk promoter sequences were cloned into the pGL4.0 luciferase reporter vector. The pRL-TK plasmid and the recombinant plasmids were co-transfected into HCC cells. Firefly and Renilla luciferase activities were successively quantified from the same sample using the Dual-Luciferase Reporter Assay System (Promega, Madison, WI, USA) to assess luciferase activity. Firefly luciferase activity was normalized to Renilla luciferase, and results were expressed as fold changes relative to the control.

### Chromatin immunoprecipitation (ChIP) assay

Following the manufacturer's instructions, a commercial kit was used to perform the ChIP assay. Briefly, 1% formaldehyde was used to cross-link cells. Through sonication, 200–1000 bp DNA fragments were retrieved. Overnight incubation at 4 °C was performed with nonspecific IgG mouse antibody and ChIP-grade FOXO3 antibody to precipitate the chromatin. Following the extraction and purification of DNA from the chromatin complexes, qPCR was used to determine the abundance of Syk. The following were the primers for the ChIP assay: forward: 5’-CCAATAACCCTGGAAGCAGA-3' and reverse: 5’-ATAACACTGGTTGGCAGC −3'.

### Sample preparation and iTRAQ labeling

Samples from the Troxerutin and DMSO groups were pooled in equal volumes. High-abundance proteins were removed using a Human 14 Multiple Affinity Removal System (Agilent, Santa Clara, CA, USA). Protein concentrations were determined with a Bradford Protein Assay Kit (Bio-Rad, Hercules, CA, USA). For each sample, 100 µg of protein was reduced, alkylated, and digested with trypsin according to the manufacturer’s instructions. The resulting peptides were labeled with an 8-plex iTRAQ reagent kit (Applied Biosystems, USA). The labeled peptides were then combined, desalted using a Sep-Pak Vac C18 column (Waters, USA), and lyophilized.

### iTRAQ quantitative proteomics and DEPs identification

DEPs were defined as those with a fold change ≥ 1.2 for upregulated proteins or ≤ 0.83 for downregulated proteins, together with a *P*-value < 0.05. Proteins were further required to contain at least two unique peptides and have a confidence interval > 95% to ensure reliable quantification. This threshold has been widely applied in iTRAQ-based proteomic studies (Tian et al. 2019) and balances the detection of biologically meaningful changes with the technical variability inherent to isobaric labeling.

### Animals

The Guangdong Medical Laboratory Animal Center (Guangzhou, China) provided the male BALB/c nude mice (6 weeks old), which were kept in a 12-h light/dark cycle under specified pathogen-free (SPF) conditions. Approximately 3 × 10⁶ MHCC-97H cells were suspended in 100 µL PBS and subcutaneously injected into mice (n = 5 per group, based on an exploratory design and the principle of minimizing animal use). In the troxerutin treatment groups, mice received intravenous injections of troxerutin at 50, 100, or 200 mg/kg daily for 28 days, while control mice received normal saline. These doses were selected from our preliminary animal experiments, in which different dose levels were tested for tolerability and antitumor effects. Tumor dimensions were measured weekly, and tumor volume was calculated using the formula: length × width^2^ × 0.5. Four weeks after cell injection, mice were sacrificed, tumors were excised, and tumor weights were recorded. Growth curves were plotted from measured tumor volumes. For further analysis, the tumor samples were split: one part was snap-frozen in liquid nitrogen for future tests, and another part was fixed in neutral formalin for histology. The Animal Ethics Committee of Guangzhou University of Chinese Medicine approved all procedures.

### Immunohistochemistry (IHC) assay

Ki‑67, CK19, and AFP protein expression in tumor tissues was evaluated by IHC. Tumor samples were fixed in formalin, embedded in paraffin, and sectioned at 2 µm thickness onto positively charged slides. Sections were treated with 3% H₂O₂ for antigen retrieval and blocked with 3% BSA. Primary antibodies against Ki‑67 (1:5000, Proteintech Cat# 27,309–1-AP, RRID:AB_2756525), CK19 (1:20,000, Proteintech Cat# 10,712–1-AP, RRID:AB_2133325), and AFP (1:500, Proteintech Cat# 14,550–1-AP, RRID:AB_2223933) were applied for 20 min, followed by three PBS washes. After 30 min of room temperature secondary antibody incubation, sections were again washed with PBS. Staining was developed with 100 µL DAB solution for 10 min, and results were examined microscopically. Whole slides were scanned using a Pannoramic 250 FLASH scanner, and three randomly selected, non‑overlapping fields were analyzed to determine the positive area ratio through ImageJ software.

### Statistical analysis

The data were processed and analyzed with SPSS Statistics (IBM, Armonk, NY, USA), and graphical representations were created via GraphPad Prism 8. Statistical significance was determined through Student’s t test or one-way ANOVA followed by Tukey’s post hoc test. All experiments were performed at least three times. Data are presented as mean ± standard deviation (SD).

## Results

### Troxerutin suppresses malignant phenotypes in HCC *in vitro*

**Fig. 1:**
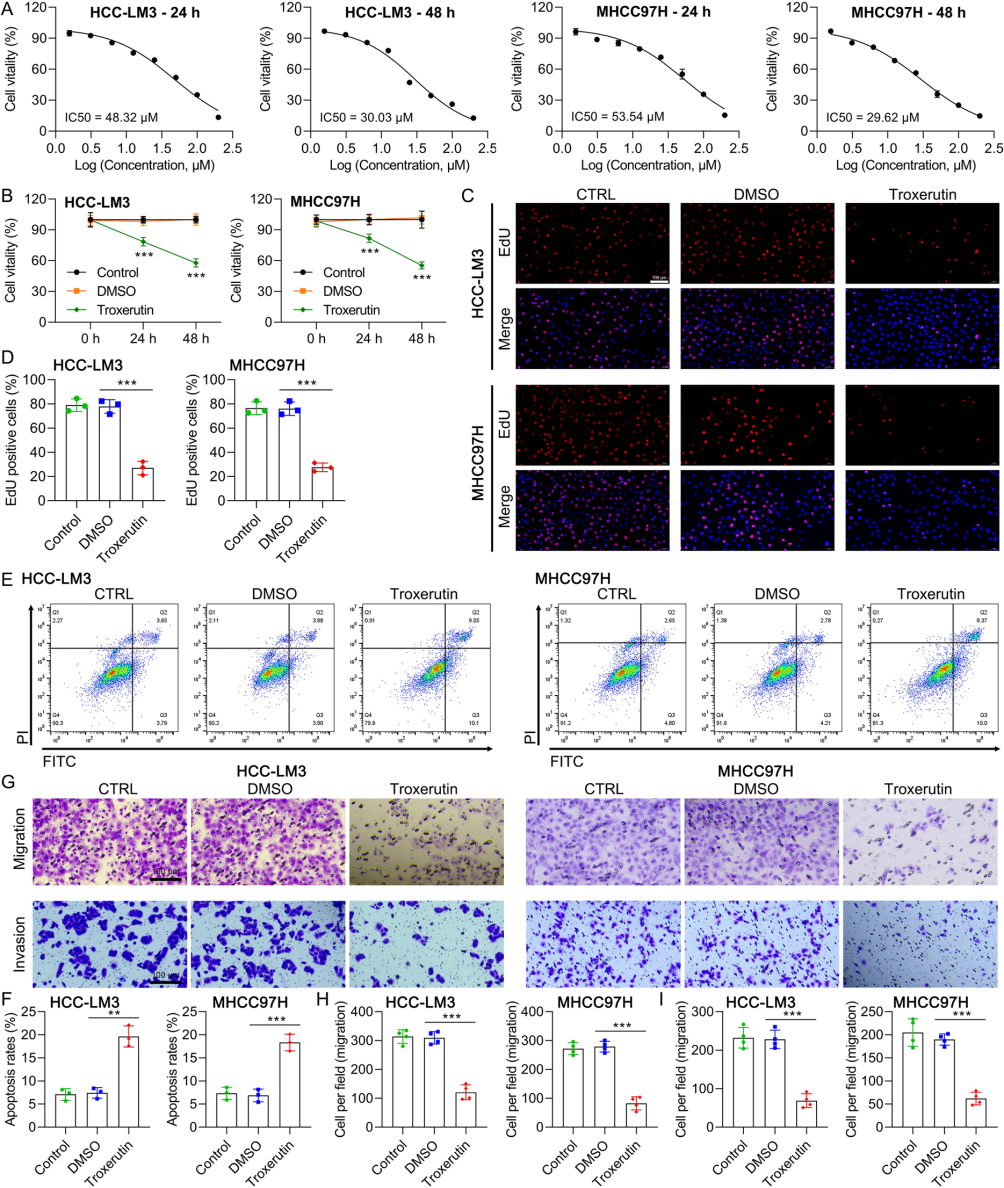
Troxerutin inhibits malignant phenotypes of HCC cells *in vitro*. (**A**) Cell viability was assessed using the CCK‑8 assay. (**B–D**) Proliferation of HCC‑LM3 and MHCC‑97H cells following troxerutin treatment was evaluated by CCK‑8 and EdU assays. (**E–F**) Apoptosis in troxerutin‑ or DMSO‑treated HCC‑LM3 and MHCC‑97H cells was analyzed by flow cytometry. (**G–I**) The effects of troxerutin on cell migration and invasion were examined using Transwell assays. Representative images (100× magnification) and corresponding quantitative analyses are shown. Three separate experiments (*n* = 3) are used to present the data as mean ± SD. **P < 0.01, ***P < 0.001

In order to assess the impact of troxerutin on highly metastatic HCC cells (HCC‑LM3 and MHCC‑97H) and determine appropriate treatment concentrations for subsequent experiments, cell viability was evaluated using the CCK‑8 assay, and the IC₅₀ values were calculated. Troxerutin treatment for 24 h and 48 h reduced cell viability in a dose‑dependent manner (Fig. 1A), and an appropriate concentration was selected for subsequent experiments. Using CCK-8, EdU, and flow cytometry assays, the effect of troxerutin on the proliferation and apoptosis of HCC-LM3 and MHCC-97H cells was assessed (Fig. 1B–F). CCK‑8 results showed that 20 μM troxerutin inhibited cell proliferation compared with controls (Fig. 1B), consistent with reduced EdU incorporation observed in EdU assays (Fig. 1C–D). Moreover, flow cytometry analysis revealed that 20 μM troxerutin significantly increased apoptosis in HCC cells relative to both control and DMSO‑treated groups (Fig. 1E–F). In line with these results, Transwell migration and invasion assays showed that troxerutin significantly reduced the metastatic potential of HCC‑LM3 and MHCC‑97H cells, as indicated by their decreased ability to traverse the membrane matrix (Fig. 1G–I). Collectively, these findings suggest that troxerutin inhibits malignant phenotypes of HCC cells in vitro.

### Troxerutin inhibits HCC cell stemness *in vitro*

**Fig. 2:**
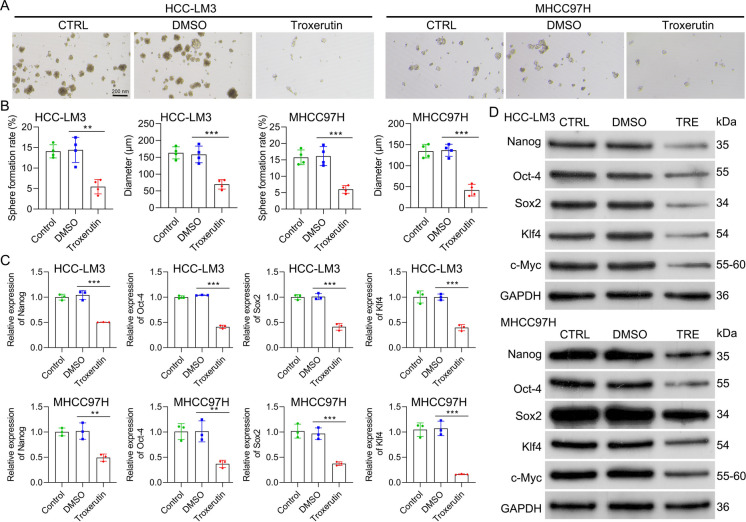
Troxerutin reduces stemness in HCC‑LM3 and MHCC‑97H cells. (**A**) Illustration of tumor spheroid formation in HCC cells treated with vehicle control, DMSO, or troxerutin (sphere formation assay, *in vitro*). (**B**) Quantification of sphere number and diameter in troxerutin‑treated HCC cells compared with controls. (**C–D**) Expression of stemness markers Sox2, Nanog, Klf4, c‑Myc, and OCT4 was evaluated by western blotting. Relative protein levels were quantified using ImageJ and presented as bar graphs. Mean ± SD, *n* = 3 independent experiments. **P < 0.01, ***P < 0.001

A spheroid formation assay was subsequently conducted. As shown in Fig. 2A–B, treatment with 20 μM troxerutin significantly decreased both the diameter and formation rate of spheres compared with controls. Stem cell markers Sox2, Nanog, Klf4, c‑Myc, and OCT4 were further examined. According to qPCR and Western blot tests, troxerutin administration substantially lowered the expression of stemness markers (Sox2, Nanog, Klf4, c-Myc, and OCT4) at both the mRNA and protein levels (Fig. 2C-D and Figure S1). In vivo limiting dilution assays were not performed, which is a limitation of the current study. These results showed that troxerutin reduced the stemness of HCC-LM3 and MHCC-97H cells.

### DEPs and KEGG pathway analysis in troxerutin‑treated HCC cells

**Fig. 3:**
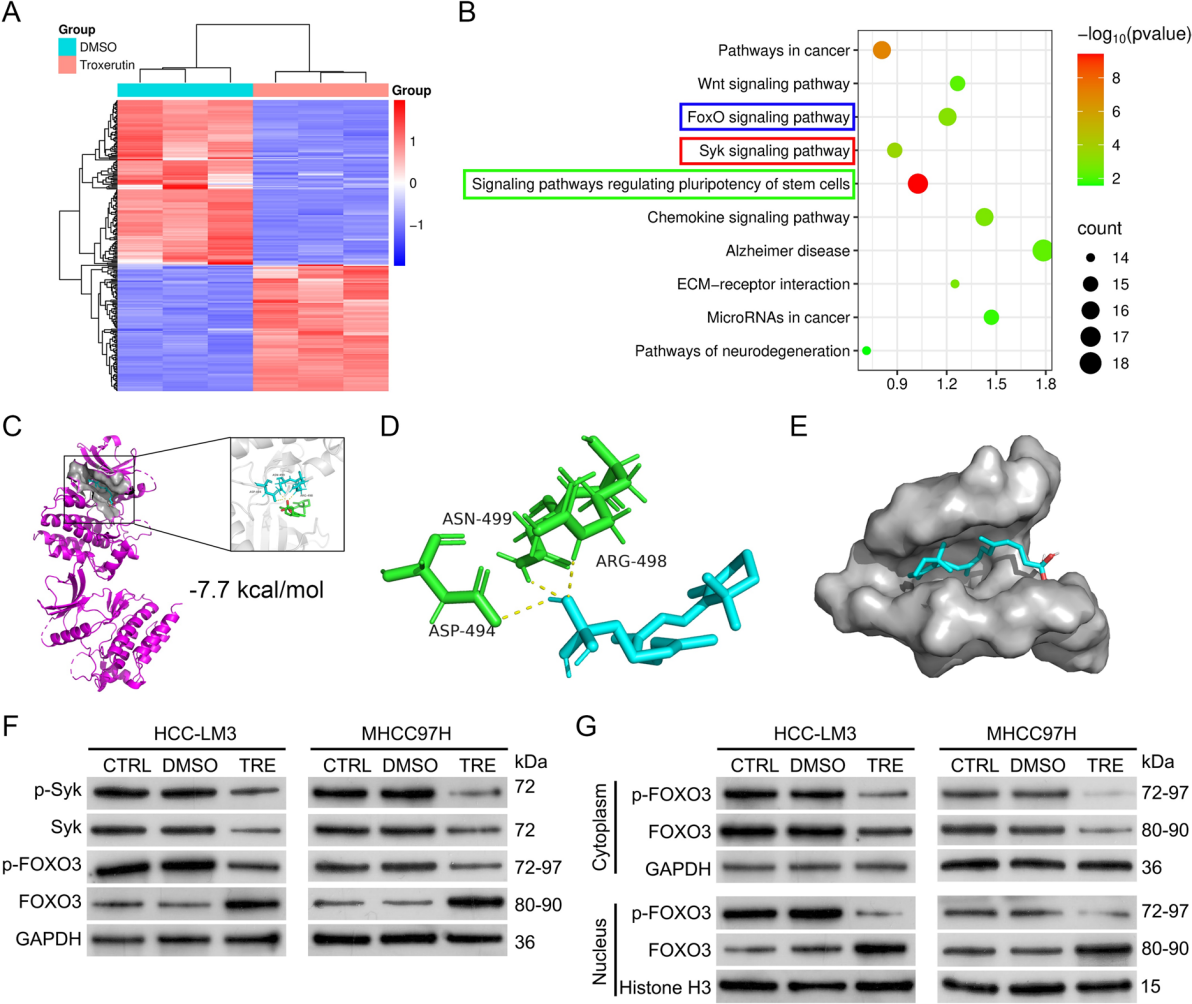
Identification of DEPs and KEGG pathway analysis in HCC cells treated with troxerutin. **(A)** Heatmap showing protein expression profiles in troxerutin-treated (10 μg/mL) and DMSO control groups. Each row represents a protein and each column a sample. Red indicates higher expression; blue indicates lower expression. **(B)** KEGG pathway enrichment analysis of DEPs. Dot size indicates the number of DEPs mapped to each pathway; color represents –log10(P-value). **(C)** Molecular docking of troxerutin with Syk. Binding energy was calculated as –7.7 kcal/mol. **(D)** Detailed view of the active pocket showing hydrogen-bond interactions between troxerutin and Syk residues ASN‑499, ARG‑498, and ASP‑494. **(E)** Surface representation of the troxerutin–Syk complex showing the ligand binding site. **(F)** Western blot analysis of p‑Syk, Syk, p‑FOXO3, and FOXO3 in HCC‑LM3 and MHCC‑97H cells. **(G)** Western blot analysis of cytoplasmic and nuclear fractions. p‑FOXO3 and FOXO3 were detected in both compartments, with GAPDH and Histone H3 as cytoplasmic and nuclear markers, respectively

Using LC‑MS/MS combined with iTRAQ, a thorough proteome study was carried out. A total of 276 DEPs were found using strict thresholds (≥ 1.2‑fold increase or ≤ 0.83‑fold decrease, p < 0.05), with 148 downregulated and 128 upregulated in the troxerutin-treated group as compared to controls (Fig. 3A). To further explore the molecular mechanisms, KEGG pathway enrichment analysis was conducted on the DEPs. The FOXO signaling route, the Syk signaling pathway, and the pathways controlling stem cell pluripotency were the ones that were more abundant in the troxerutin group in comparison to the DMSO group (Fig. 3B). To further identify the troxerutin’s function in Syk/FOXO3 pathway, the molecular docking program was firstly employed to investigate the binding potential of troxerutin to Syk. The optimal binding conformation of the troxerutin-Syk complexes was presented in Fig. 3C, with a calculated binding energy of −7.7 kcal/mol, which predicted good binding activity to Syk, and the active pocket formed by the optimal docking of receptor and ligand after visualization was relatively stable (Fig. 3D and E). To validate these results, western blot analysis was performed to assess p‑Syk, Syk, FOXO3, and p‑FOXO3 protein levels. The proteomic findings showed that the troxerutin-treated group had lower levels of p-Syk, Syk, and p-FOXO3 expression than the control group (Fig. 3F and Figure S2A). FOXO3 was upregulated in the troxerutin group, suggesting that troxerutin may regulate Syk dephosphorylation in HCC cells. Thus, we hypothesize that Syk dephosphorylation-mediated FOXO3 activation increases its expression and nuclear accumulation. To further test this hypothesis, cytoplasmic and nuclear fractions of HCC‑LM3 and MHCC‑97H cells were isolated, and p‑FOXO3 and FOXO3 levels were analyzed by western blot. The cytoplasmic and nuclear fractions were identified by GAPDH and Histone H3, respectively. Compared with controls, troxerutin treatment increased FOXO3 levels in the nucleus while reducing its cytoplasmic expression. Similarly, phosphorylated FOXO3 (p‑FOXO3) was significantly decreased in both fractions (Fig. 3G, Figure S2B, and Figure S2C). These results indicate that Syk dephosphorylation–mediated activation of FOXO3 promotes its nuclear accumulation. Collectively, these findings suggest that the FOXO3/Syk axis is a primary target of troxerutin and was therefore selected for further investigation.

### Downregulation of Syk impedes the malignant phenotypes and cell stemness of HCC *in vitro*

**Fig. 4:**
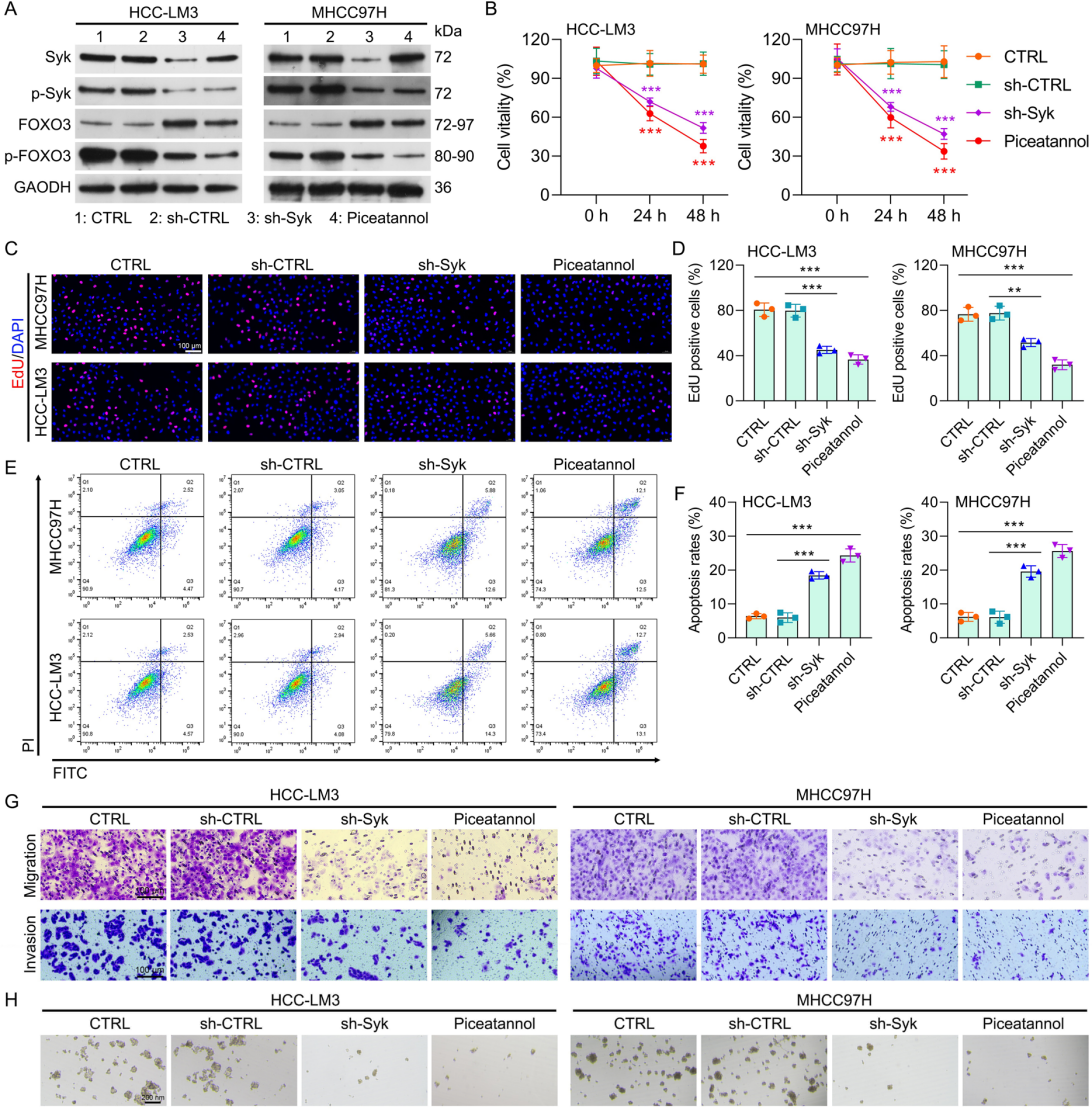
Downregulation of Syk inhibits malignant phenotypes in HCC cells. (**A**) Western blot verification of Syk inhibition by shRNA or piceatannol in HCC‑LM3 and MHCC‑97H cells. (**B–D**) EdU and CCK‑8 assays showing reduced proliferation following Syk downregulation. (**E–F**) Flow cytometry analysis of apoptosis in CTRL, sh‑CTRL, sh‑Syk, and piceatannol treatment groups. (**G**) Transwell assays demonstrating decreased migration and invasion after Syk suppression; representative images shown at 100× magnification. (**H**) Representative pictures from sphere formation assays illustrating reduced spheroid size and number upon Syk downregulation. Data are mean ± SD, *n* = 3 independent experiments. **P < 0.01, ***P < 0.001

Based on these findings, we hypothesized that Syk and FOXO3 play pivotal roles in HCC development and progression. To further investigate the physiological function of Syk, a series of cellular experiments was performed in HCC‑LM3 and MHCC‑97H cells. Western blot analysis confirmed the silencing efficiency of Syk‑specific shRNAs as well as the inhibitory effect of piceatannol, a selective Syk inhibitor. Both sh‑Syk and piceatannol reduced p‑Syk expression; however, only sh‑Syk significantly decreased total Syk levels, while piceatannol did not alter Syk protein expression (Fig. 4A and Figure S3). We also observed that both sh-Syk and piceatannol increased FOXO3 protein expression while reducing p-FOXO3 levels. Syk downregulation's effect on cell viability was then evaluated using the CCK-8 and EdU assays. The Syk knockdown and piceatannol treatment significantly reduced cell viability in HCC‑LM3 and MHCC‑97H cells (Fig. 4B). Similarly, EdU assays demonstrated that Syk suppression also considerably inhibited cell proliferation (Fig. 4C–D). Apoptosis was subsequently evaluated by flow cytometry, as illustrated in Fig. 4E–F. Syk downregulation increased apoptosis in HCC cells compared with both control groups. Accordingly, Syk inhibition dramatically decreased the metastatic potential of HCC-LM3 and MHCC-97H cells, as demonstrated by their reduced ability to pass through the membrane matrix, according to Transwell migration and invasion studies (Fig. 1G and Figure S4A-B). Spheroid formation assays demonstrated that Syk downregulation significantly decreased both the size and number of spheres compared with controls (Fig. 4H and Figure S4C-D). Stem cell marker expression was further examined. Syk downregulation led to decreased levels of Sox2, Nanog, Klf4, c‑Myc, and OCT4 (Figure S4E and Figure S5A). Furthermore, Syk suppression reduced p‑FOXO3 expression in both the cytoplasm and nucleus of HCC cells, while promoting increased nuclear accumulation of FOXO3 (Figure S4F, Figure S5B, Figure S5C). These findings indicate that Syk downregulation suppresses the malignant behavior and stemness of HCC cells, likely through a cascade in which Syk dephosphorylation facilitates FOXO3 dephosphorylation, promoting its nuclear accumulation and transcriptional activation.

### Upregulation of Syk reverses troxerutin's inhibitory effects on malignant phenotypes in HCC cells

**Fig. 5:**
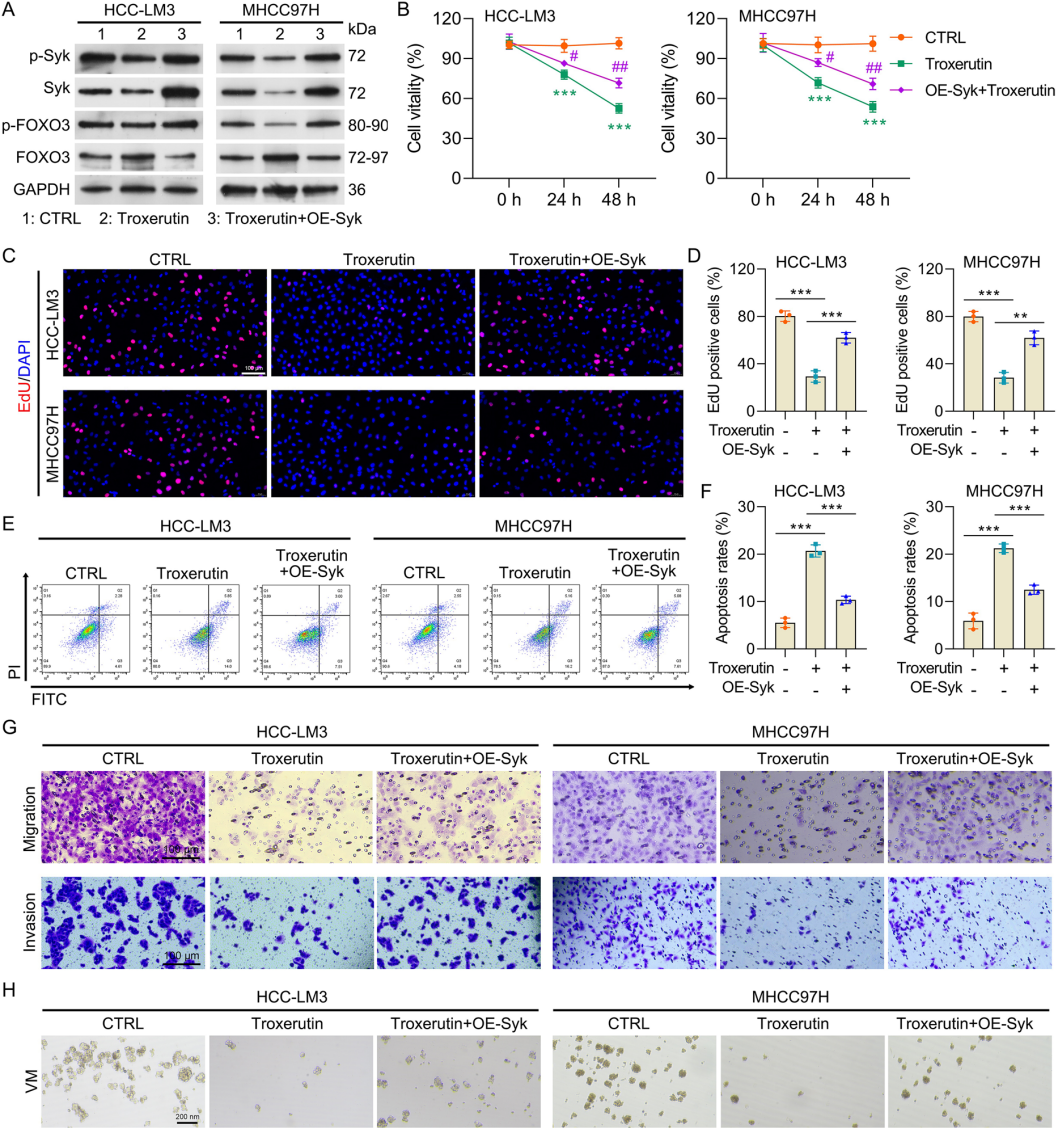
Syk overexpression counteracts troxerutin‑mediated inhibition of malignant phenotypes in HCC cells. (**A**) Western blot analysis of p‑Syk, Syk, p‑FOXO3, and FOXO3 expression in HCC‑LM3 and MHCC‑97H cells across three groups: CTRL, troxerutin‑treated, and troxerutin + Syk overexpression (OE‑Syk), compared with CTRL, ***P < 0.001; compared with Troxerutin, #*P* < 0.05, ##*P* < 0.01. (**B–D**) CCK‑8 (**B**) and EdU (**C, D**) assays evaluating proliferation in the 3 experimental groups. (**E–F**) Flow cytometry analysis assessing apoptosis in the same cohorts. **(G)** Transwell assays evaluating migration (top) and invasion (bottom) of HCC‑LM3 and MHCC‑97H cells. **(H)** Vasculogenic mimicry (VM) assay showing tube-like structures in the three experimental groups. Data are mean ± SD, *n* = 3 independent experiments. **P < 0.01, ***P < 0.001

To ascertain if troxerutin's inhibitory effects on HCC‑LM3 and MHCC‑97H cells were Syk-dependent, rescue studies were carried out. Cells were treated with troxerutin alone or co‑treated with troxerutin and a Syk overexpression vector. Co‑treatment reversed the troxerutin-induced reduction of p‑Syk and Syk levels in both HCC‑LM3 and MHCC‑97H cells. Similarly, the increase in FOXO3 expression and the decrease in p‑FOXO3 levels induced by troxerutin were reversed by Syk overexpression (Fig. 5A and Figure S6). Cell proliferation was evaluated using CCK‑8 and EdU assays. Troxerutin significantly inhibited proliferation in both HCC‑LM3 and MHCC‑97H cells, whereas Syk overexpression partially reversed this effect in both cell lines (Fig. 5B-D). Flow cytometry analysis confirmed that Syk upregulation inhibited troxerutin-induced apoptotic cell death in both HCC‑LM3 and MHCC‑97H cells (Fig. 5E-F). Furthermore, Syk overexpression significantly reduced the inhibitory effects of troxerutin on cell migration and invasion, with consistent results in HCC‑LM3 and MHCC‑97H cells according to Transwell assays (Fig. 5G and Figure S7A-B). Results from the spheroid formation assay revealed that Syk overexpression significantly reversed troxerutin-mediated suppression of spheroid formation, as evidenced by restored spheroid diameter and number in both cell lines (Fig. 5H and Figure S7C-D). Stem cell marker analysis showed that Syk overexpression increased the levels of Sox2, Nanog, Klf4, c‑Myc, and OCT4 (Figure S7E and Figure S8A). Moreover, Syk upregulation reversed the troxerutin-induced increase of nuclear FOXO3 and the decrease of cytoplasmic FOXO3, while elevating p‑FOXO3 levels in both compartments, thereby counteracting the effect of troxerutin (Figure S7F, Figure S8B, and Figure S8C). These results indicate that upregulation of Syk reverses troxerutin's inhibitory effects on malignant phenotypes in HCC cells and further demonstrate that the Syk/FOXO3 axis constitutes the critical pathway regulated by troxerutin.

### FOXO3 directly binds to the SYK promoter and suppresses its transcription

**Fig. 6:**
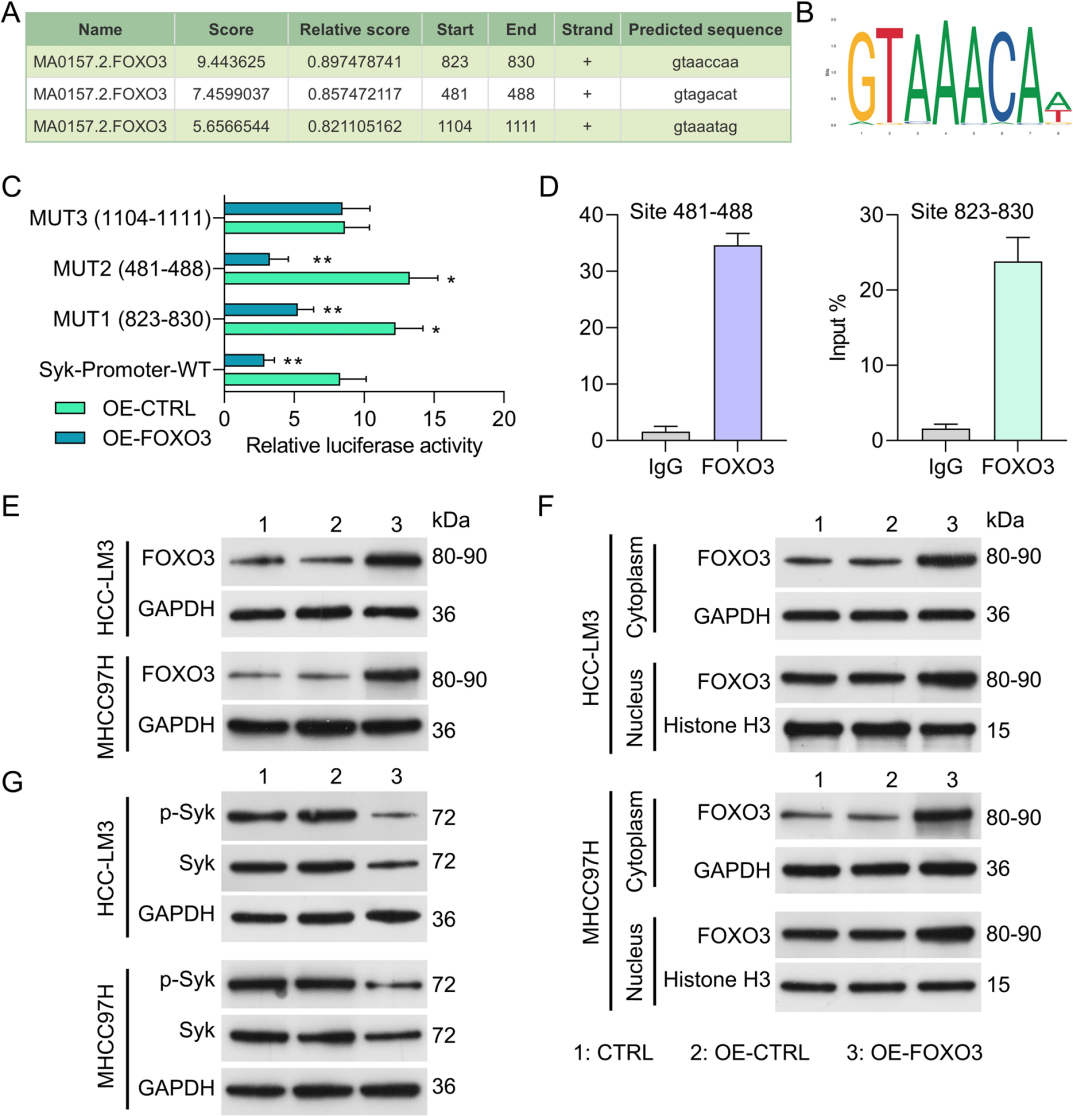
FOXO3 binding to SYK prom oter analyzed by luciferase assay, ChIP, and Western blot. (**A**) Predicted FOXO3 binding site within the Syk promoter region and the top three predicted binding sequences. (**B**) JASPAR database output showing FOXO3 consensus binding motifs. (**C**) A dual-luciferase reporter assay was performed on HCC cells transfected with OE-FOXO3 or OE-CTRL and co-transfected with either wild-type or mutant promoter constructs. (**D**) ChIP analysis confirming the binding of FOXO3 to the Syk promoter in HCC cells. (**E**) Western blot verification of FOXO3 overexpression in OE‑FOXO3 and OE‑CTRL groups. (**F**) Subcellular localization of FOXO3 detected by Western blot following nuclear and cytoplasmic protein fractionation; GAPDH and Histone H3 served as cytoplasmic and nuclear markers, respectively. (**G**) Syk and p-Syk expression in the designated HCC cell types as determined by Western blot analysis. Data are mean ± SD, *n* = 3 independent experiments. *P < 0.05, **P < 0.01

To investigate the molecular mechanism by which FOXO3 regulates Syk, the JASPAR database was used to predict potential FOXO3 binding sites within the Syk promoter region, revealing a putative binding site (Fig. 6B). Figure 6A displayed the motifs with the highest correlation after we predicted the sequence of the upstream region of the Syk promoter and FOXO3 binding site. To determine if FOXO3 regulates Syk transcriptionally, ChIP and dual-luciferase reporter assays were performed. A mutation was introduced at the predicted binding site to assess its importance for FOXO3 interaction. The dual-luciferase assay demonstrated that this sequence within the Syk promoter is essential for FOXO3 binding and regulation of promoter activity (Fig. 6C). ChIP analysis confirmed significant enrichment of FOXO3 at the Syk promoter region in HCC cells (Fig. 6D). FOXO3 was then overexpressed in HCC-LM3 and MHCC-97H cells, with successful overexpression verified by western blotting (Fig. 6E and Figure S9A). Cytoplasmic and nuclear fractions were isolated from these cells, and FOXO3 levels were assessed by Western blot. FOXO3 expression increased in both the cytoplasmic fraction, marked by GAPDH, and the nuclear fraction, indicated by Histone H3. However, nuclear accumulation was less pronounced compared to the marked increase in the cytoplasm (Fig. 6F and Figure S9B), demonstrating that FOXO3 is upregulated in both compartments but predominantly localized in the cytoplasm. Finally, Syk and p-Syk expression were measured across three groups (CTRL, OE-CTRL, and OE-FOXO3). Western blot results showed significant inhibition of both Syk and p-Syk upon FOXO3 overexpression, indicating that FOXO3 acts as a negative regulator of Syk activation (Fig. 6G and Figure S9C). Overall, these findings demonstrate that FOXO3 negatively regulates Syk transcription by directly binding to a specific site within the Syk promoter.

### Overexpression of FOXO3 impedes the malignant and stemness of HCC phenotypes *in vitro*

**Fig. 7:**
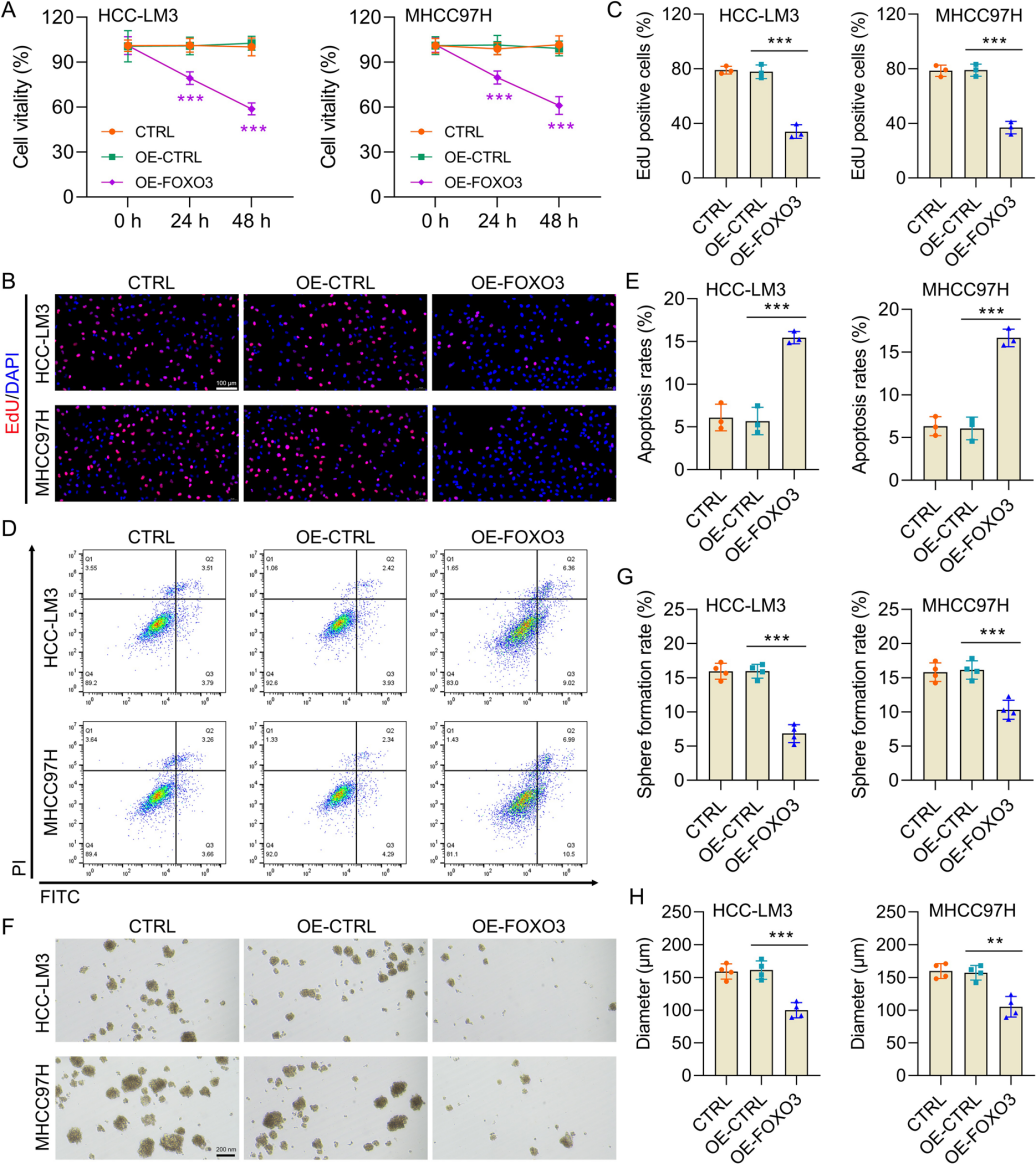
*In vitro*, FOXO3 overexpression inhibits the malignant characteristics of HCC cells. (**A–C**) FOXO3 overexpression substantially suppresses the proliferation of HCC-LM3 and MHCC-97H cells, according to CCK-8 and EdU experiments. (**D–E**) Flow cytometric analysis showing increased apoptosis in HCC cells transfected with the FOXO3 overexpression vector compared to the control vector. (**F**) Representative images of the *in vitro* sphere formation assay in control and FOXO3‑overexpressing HCC cells. (**G–H**) Quantification of sphere number and diameter in FOXO3‑overexpressing versus control cells. Data are mean ± SD, *n* = 3 independent experiments. **P < 0.01, ***P < 0.001

The present study overexpressed FOXO3 in MHCC-97H and HCC-LM3 cells to confirm its function. This overexpression decreased the probability of metastasis (Figure S10A–D), induced apoptosis (Fig. 7D–E), and inhibited cell proliferation (Fig. 7A–C). Further, a spheroid formation assay was carried out. As shown in Fig. 7F-H, FOXO3 overexpression significantly reduced the spheres in both diameter and number compared with the control groups. Furthermore, FOXO3 overexpression led to a reduction in cancer stemness markers (Figure S10E and Figure S11). These findings indicate that FOXO3 suppresses the malignant behavior and stemness of HCC‑LM3 and MHCC‑97H cells in vitro.

### Troxerutin suppresses HCC malignancy by modulating FOXO3

**Fig. 8:**
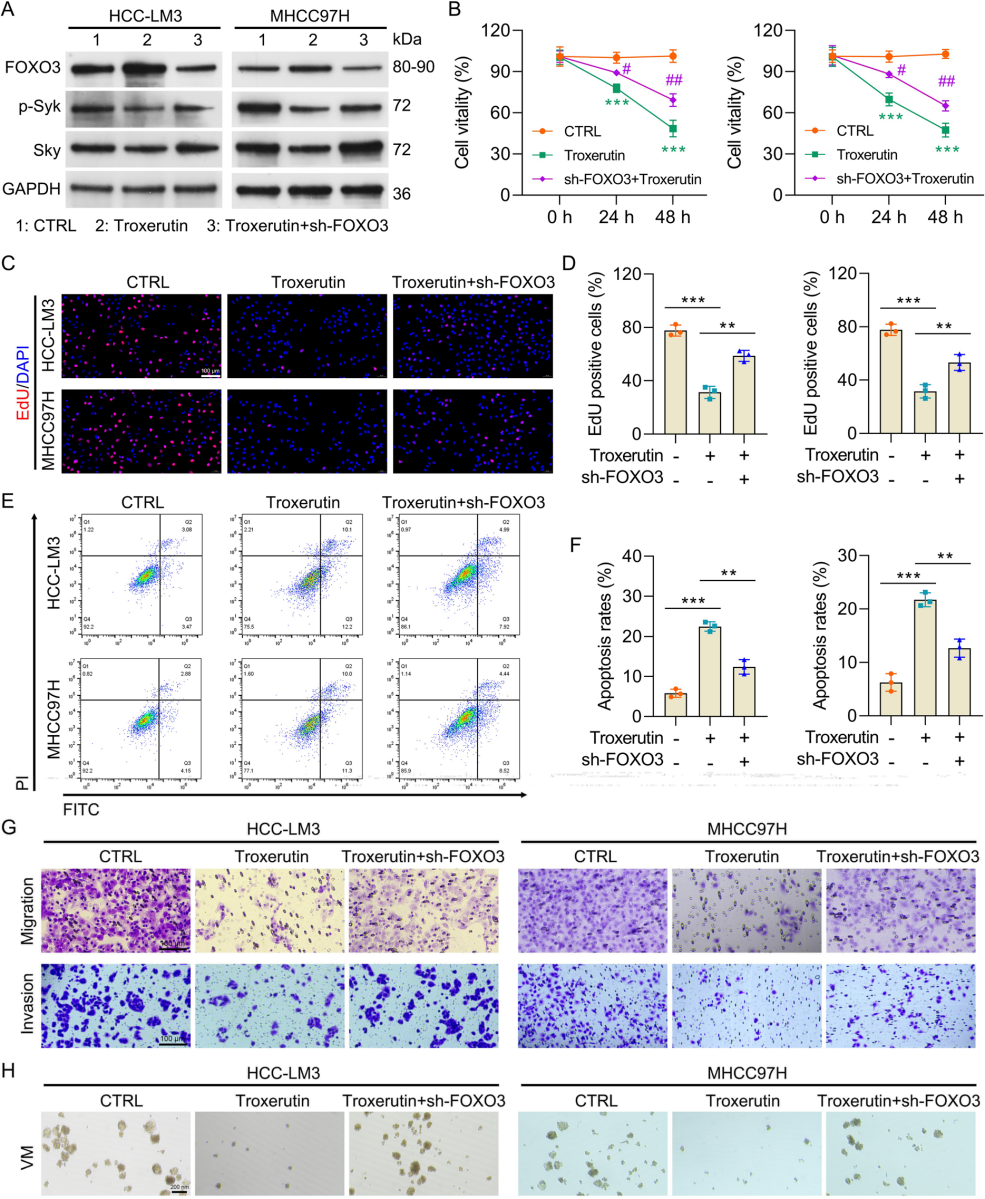
Troxerutin suppresses HCC malignancy through modulation of FOXO3. (**A**) Western blot analysis of FOXO3, p‑Syk, and Syk protein levels in HCC‑LM3 and MHCC‑97H cells treated with troxerutin alone or in combination with sh‑FOXO3, compared with CTRL, ***P < 0.001; compared with Troxerutin, #*P* < 0.05, ##*P* < 0.01. (**B**) CCK‑8 assays evaluating whether FOXO3 knockdown reverses the antiproliferative effect of troxerutin. (**C–D**) EdU staining assessing the impact of FOXO3 knockdown on troxerutin‑mediated suppression of proliferation. (**E–F**) Flow cytometric analysis examining whether FOXO3 knockdown counteracts troxerutin‑induced apoptosis. (**G**) Transwell migration and invasion assays showing restoration of motility and invasiveness following FOXO3 knockdown (images = 100 × magnification power). (**H**) Representative images from the in vitro sphere formation assay. Data are mean ± SD, *n* = 3 independent experiments. **P < 0.01, ***P < 0.001

To ascertain whether the inhibitory malignancy effect of troxerutin is mediated through regulating FOXO3, we administered troxerutin to HCC cells with FOXO3-specific shRNA. Western blot analysis revealed that p‑Syk and Syk levels were reduced in the troxerutin‑treated group compared with controls, an effect markedly reversed by sh‑FOXO3. Consistent with earlier results, troxerutin increased FOXO3 expression in HCC cells, which was effectively suppressed by FOXO3 knockdown (Fig. 8A and Figure S12). The treatment with sh-FOXO3 effectively restored their proliferative capacity (Fig. 8B-D), reduced cell apoptosis (Fig. 8E-F), and suppressed their metastatic potential (Fig. 8G and Figure S13A-D). Importantly, FOXO3 knockdown (sh-FOXO3) significantly reversed troxerutin's effects, restoring both the number and diameter of tumor spheres to control levels (Fig. 8H and Figure S13C-D). Consistent with this phenotypic rescue, sh-FOXO3 treatment abrogated troxerutin-mediated suppression of cancer stem cell markers (Figure S13E and Figure S14). These findings establish FOXO3 as the critical mediator of troxerutin's anti-tumor activity in HCC.

### FOXO3 facilitates HCC malignancy by interacting with Syk

**Fig. 9:**
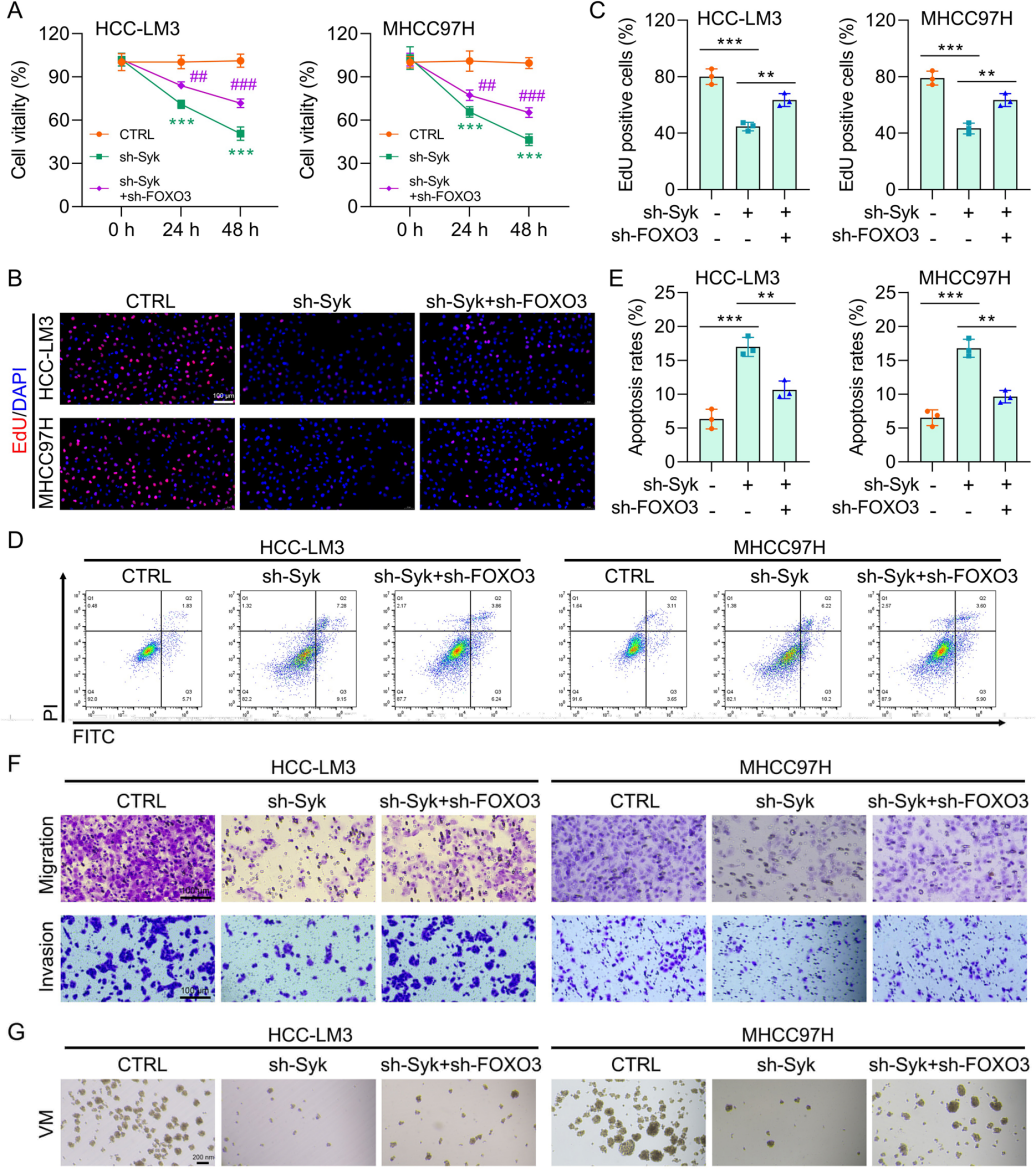
FOXO3 promotes HCC malignancy via interaction with Syk. (**A**) CCK‑8 and (**B–C**) EdU assays evaluating the proliferative capacity of HCC‑LM3 and MHCC‑97H cells treated with sh‑FOXO3 in the context of Syk knockdown, compared with CTRL, ***P < 0.001; compared with sh-Syk, ##*P* < 0.01, ###*P* < 0.001. (**D–E**) Flow cytometric analysis examining whether FOXO3 knockdown reverses sh‑Syk–induced apoptosis. (**F**) The impact of FOXO3 knockdown on the decreased motility and invasiveness caused by Syk silencing was evaluated using transwell migration and invasion assays (images = 100 × magnification power). (**G**) Representative images from the in vitro sphere formation assay. Data are mean ± SD, *n* = 3 independent experiments. **P < 0.01, ***P < 0.001

We assessed the Syk-FOXO3 functional relationship by expressing sh-FOXO3 in Syk-deficient HCC cells. The treatment with sh-FOXO3 effectively restored their proliferative capacity (Fig. 9A-C), reduced cell apoptosis (Fig. 9D-E), and enhanced their metastatic potential (Fig. 9F and Figure S16A-B). Furthermore, sh-FOXO3 supplementation markedly restored both the number and size of tumor spheres (Fig. 9G and Figure S16C-D). Western blot analysis showed that Syk knockdown reduced Syk and p-Syk expression but increased FOXO3 and p-FOXO3 expression in both HCC-LM3 and MHCC97H cells, while these changes were partially reversed by sh-FOXO3 co-transfection (Figure S15). In particular, FOXO3 knockdown resulted in a substantial elevation of cancer stemness markers in comparison to the sh-Syk group (Figure S16E and Figure S17). This demonstrates that FOXO3 facilitates HCC malignancy by interacting with Syk.

### Troxerutin inhibits HCC growth in mice dose‑dependently

**Fig. 10:**
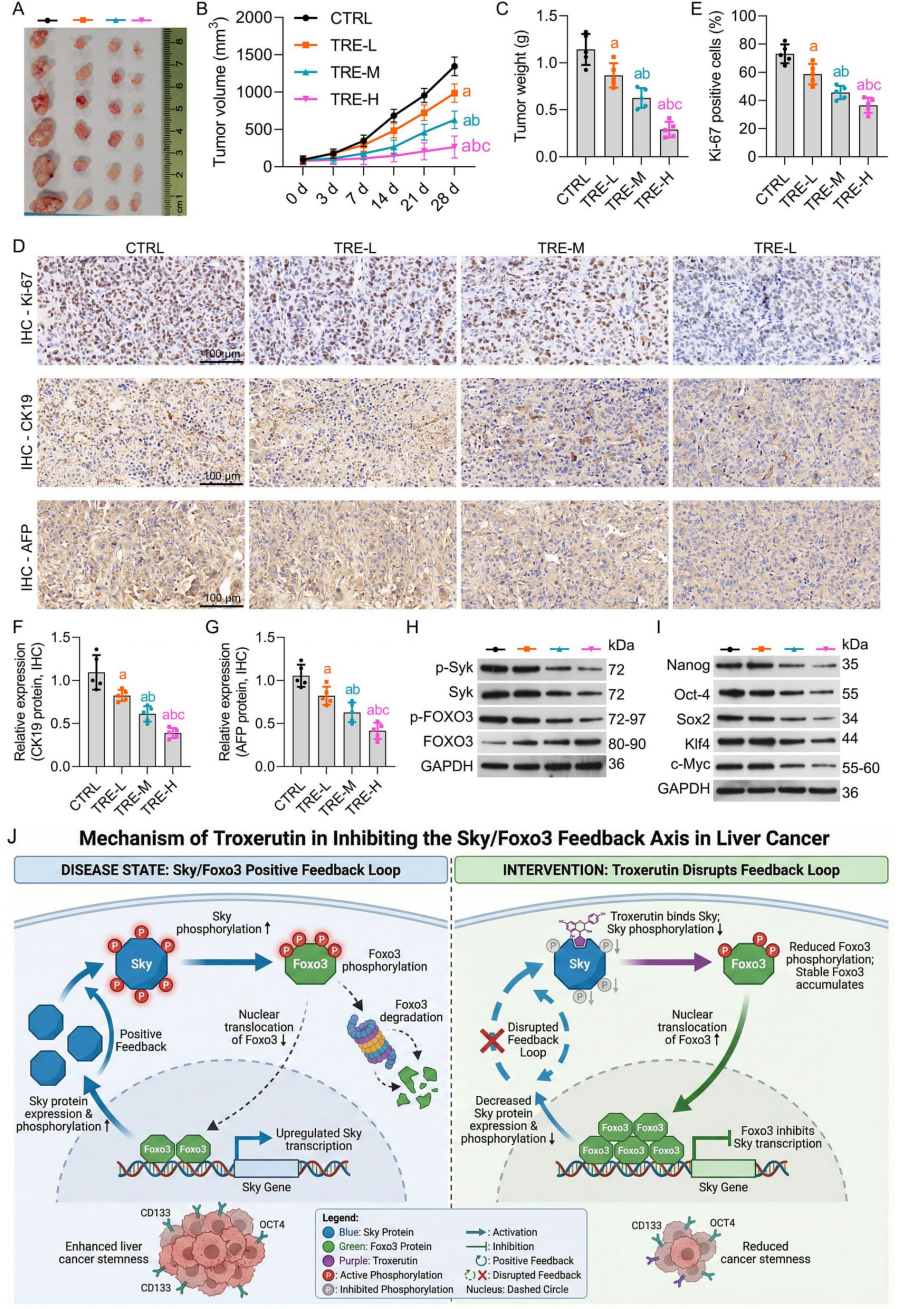
Troxerutin suppresses HCC growth in murine models in a dose-dependent manner. Tumor weight at the endpoint of the study. (A) Representative tumor images from each treatment group. (**B–C**) Statistical analysis of tumor volume and tumor weight (n = 3, mean ± SEM). (**D**) Immunohistochemical staining of Ki67, CK19, and AFP in tumor sections; scale bar = 50 μm. (**E–G**) Quantitative analysis of IHC results. (**H**) Western blot analysis of Syk, p‑Syk, p‑FOXO3, and FOXO3 protein levels. (**I**) Western blot analysis of stemness markers Sox2, Nanog, Klf4, c‑Myc, and OCT4. (**J**) Troxerutin suppresses HCC progression by targeting the FOXO3/Syk signaling axis, reducing the stem‑like properties of HCC cells. Data are mean ± SD, *n* = 3 independent experiments. Compared with CTRL, a *P* < 0.05, compared with TRE-L, b *P* < 0.05, compared with TRE-M, c *P* < 0.05

To evaluate the in vivo effects of troxerutin on HCC tumor growth, a xenograft model was established by implanting MHCC‑97H cells into male BALB/c nude mice, which were then treated with different concentrations of troxerutin. Compared with the CTRL groups, troxerutin treatment restrained the growth of the subcutaneous tumor (Fig. 10A-C). Troxerutin administration dose-dependently inhibited tumor progression, as evidenced by significant reductions in both tumor weight and volume. Furthermore, immunochemical analysis revealed a considerably proliferative (Ki67) and hepatocellular carcinoma markers (CK19, AFP) in troxerutin-treated tumor sections versus controls, with a clear dose-dependent suppression pattern (Fig. 10D-G). Moreover, these results verified the p-FOXO3, FOXO3 p-Syk, and Syk expression by WB. In line with the in vitro results, troxerutin treatment reduced p‑Syk, Syk, and p‑FOXO3 levels in a dose‑dependent manner compared with controls. On the other hand, FOXO3 expression increased progressively with higher troxerutin concentrations (Fig. 10H and Figure S18). Finally, the study of stem cell markers (Fig. 10I and Figure S18) indicated that troxerutin treatment diminished their expression in a dose-dependent fashion. These data collectively suggest that troxerutin reduces hepatocellular carcinoma growth in murine models in a dose-dependent manner.

## Discussion

HCC is the most prevalent primary liver cancer, which remains one of the deadliest cancers, and the third largest cause of mortality due to cancer worldwide (Guo et al. 2025). Although advances in surgical techniques and multidrug chemotherapy over the past three decades have modestly improved the 5‑year survival rate of HCC patients (Chen et al. 2025c), progress in developing novel therapeutic strategies has slowed in recent years (Akabane et al. 2025). This highlights the urgent need for more effective treatment approaches.

Troxerutin, a semi‑synthetic derivative of the natural bioflavonoid rutin, is abundant in familiar dietary sources including tea, coffee, cereal grains, and a variety of fruits and vegetables (Ahmadi et al. 2021). Owing to its superior water solubility relative to rutin, troxerutin exhibits markedly improved gastrointestinal absorption and retains an excellent safety profile, with no detectable cytotoxicity (Cha et al. 2014; Lee et al. 2024). Potent antioxidant, anti-inflammatory, anti-diabetic, and anti-tumor actions are only a few of the many pharmacological advantages of troxerutin (Ahmadi et al. 2021). Its anti‑cancer potential has been demonstrated in multiple studies, including research focusing on HCC. For instance, Thomas NS et al. reported that troxerutin inhibits HuH‑7 cell growth by inducing heme oxygenase‑1 (HO‑1) expression and promoting nuclear translocation of Nrf2 (Thomas et al. 2019). Other studies have shown that troxerutin reduces nuclear translocation of NF‑κB (p65) via LKBK downregulation, therefore suppressing inflammation, proliferation, and cell survival. In a separate investigation, troxerutin was found to inhibit hepatic tumorigenesis (Thomas et al. 2018) by reducing hepatic nodule formation, modulating enzymatic activity, and decreasing glutathione‑S‑transferase expression, PCNA levels, and S‑phase progression markers.

In agreement with previous reports, this study demonstrates that troxerutin inhibits malignant phenotypes of HCC in vitro and reveals a strong link between elevated Syk expression and HCC progression. In recent years, Syk overexpression has been implicated in multiple cancer types. For example, in breast cancer, elevated Syk promotes tumor cell survival, proliferation, invasion, and metastasis through diverse mechanisms (Kong et al. 2025; Zhou et al. 2024a). Syk activates key signaling pathways that drive cell growth and migration, including PI3K/AKT and MAPK/ERK (Nguyen et al. 2020; Zhou et al. 2025). In gastric cancer, Syk overexpression enhances the expression and stability of C‑type lectin‑like receptor 2 (CLEC2), thus promoting invasion and metastasis via AKT pathway activation (Wang et al. 2016). In this study, Syk downregulation suppressed the malignant behavior and stemness of HCC cells in vitro, whereas Syk upregulation counteracted the inhibitory effects of troxerutin on these malignant phenotypes. Mechanistically, western blot analysis showed that Syk dephosphorylation drives FOXO3 dephosphorylation, promoting its nuclear accumulation and transcriptional activation. This indicates that the Syk/FOXO3 axis is a key pathway targeted by troxerutin. Importantly, these findings confirmed that FOXO3 directly binds to the Syk promoter to initiate its transcription, therefore contributing to HCC malignancy through its interaction with Syk.

Forkhead box O3 (FOXO3), a key member of the FOXO transcription factor family, plays a context-dependent role in tumorigenesis, functioning as either a tumor suppressor or an oncogene depending on the cellular environment (Binte Hanafi et al. 2025). While extensively characterized as a pleiotropic regulator in various human malignancies, its precise role in HCC pathogenesis remains mechanistically unresolved and subject to ongoing debate in the field (Guan et al. 2025; Manoharan et al. 2024). Recently, Tan Y et al. identified FOXO3 as a transcription factor for lumican, demonstrating that it enhances lumican expression at the transcriptional level (Tan et al. 2024). Repaglinide inhibited the activation of the lumican/p53/p21 axis by blocking FOXO3's binding to the promoter region of lumican without silencing it (Tan et al. 2024). However, in our study, we found that overexpression of FOXO3 impedes the malignant and stemness of HCC phenotypes in vitro, and troxerutin suppresses HCC malignancy by modulating FOXO3.

The idea of CSCs, a subset of tumor cells with self‑renewal capacity and the ability to drive malignancy, is increasingly recognized as a critical mechanism underlying therapeutic resistance and recurrence (Darie et al. 2026; Wang et al. 2025a). Substantial evidence indicates that CSCs are highly tumorigenic and drug‑resistant across multiple cancer types (Oskarsson et al. 2014). In HCC, the presence of CSCs presents a major barrier to effective therapy (Ge et al. 2023; Loh et al. 2025). Targeting CSCs directly or suppressing their stemness properties could therefore offer a promising strategy to improve patient outcomes. Recent studies have identified SMYD2 as an essential epigenetic regulator that activates the BMP4/R‑SMADs/ID3 axis, enhancing stemness and promoting sorafenib resistance in HCC (Wang et al. 2025b).

Growing interest has been directed toward pathways that sustain tumor stemness, particularly the Wnt, Notch, and Hedgehog signaling pathways (Pannuti et al. 2010; Takebe et al. 2011). These findings demonstrate that troxerutin suppresses HCC progression by targeting the FOXO3/Syk signaling axis, reducing the stem‑like properties of HCC cells (Fig. 10J). This aligns with previous studies indicating that inhibition of this pathway can attenuate HCC malignancy. Moreover, our results confirm that Syk dephosphorylation enhances the nuclear translocation of FOXO3, which in turn amplifies stemness features in HCC.

In summary, this study is the first to combine proteomic profiling with experimental validation to explore the pharmacological mechanism of troxerutin in HCC. Our findings suggest that troxerutin suppresses HCC stem-like properties through the Syk/FOXO3 axis and may therefore represent a potential strategy for reducing HCC progression. However, this study also has several limitations. The in vivo xenograft experiments were performed with a relatively small sample size (n = 5 per group), reflecting the exploratory nature of the study and the principle of minimizing animal use. Potential crosstalk between the Syk/FOXO3 loop and other HCC-related pathways, such as Wnt and Notch, was not explored. In addition, whether Syk regulates FOXO3 through direct interaction or through other intermediates was not examined. Troxerutin was administered only by intravenous injection, and other clinically relevant routes were not evaluated. Future studies are needed to clarify the mechanism by which Syk regulates FOXO3 phosphorylation, for example by using Co-IP, pull-down, and proteomics-based approaches, and to further validate these findings in larger-scale animal experiments.

## Supplementary Information

Below is the link to the electronic supplementary material.

Supplementary file1 (DOCX 36223 KB)

## Data Availability

The datasets used and/or analysed during the current study are available from the corresponding author on reasonable request.

## Abbreviations

cDNA: Complementary DNA
ChIP: Chromatin immunoprecipitation
CSCs: Cancer stem cells
DAB: Diaminobenzidine
DEPs: Differentially expressed proteins
FOXO3: Forkhead box O3
GAPDH: Glyceraldehyde-3-phosphate dehydrogenase
HCC: Hepatocellular carcinoma
iTRAQ: Isobaric Tags for Relative and Absolute Quantitation
NANOG: Nanog homeobox
OCT4: POU class 5 homeobox 1
qRT-PCR: Quantitative Real-Time PCR
SOX2: SRY-box transcription factor 2
Syk: Spleen Tyrosine Kinase
