# Supplementary material for: Troxerutin suppresses the stemness of hepatocellular carcinoma via the Syk/FOXO3 feedback loop
Source: Cell Biol Toxicol. 2026 May 8;42(1):82. doi: 10.1007/s10565-026-10194-z (PMC13319154; doi:10.1007/s10565-026-10194-z)
Supplement: Supplementary file 1 — Supplementary file1 (DOCX 36223 KB) [file 10565_2026_10194_MOESM1_ESM.docx]

Supplementary Materials for

**Troxerutin suppresses the stemness of hepatocellular carcinoma via the Syk/FOXO3 feedback loop**

Shan Liu *et al.*

^*^ Corresponding author: Dr Wei Guo ([844608579@qq.com](mailto:844608579@qq.com)) and Hanrui Chen ([chenhanrui1689@gzucm.edu.cn](mailto:chenhanrui1689@gzucm.edu.cn))

**This file includes:**Figure S1 to Figure S18


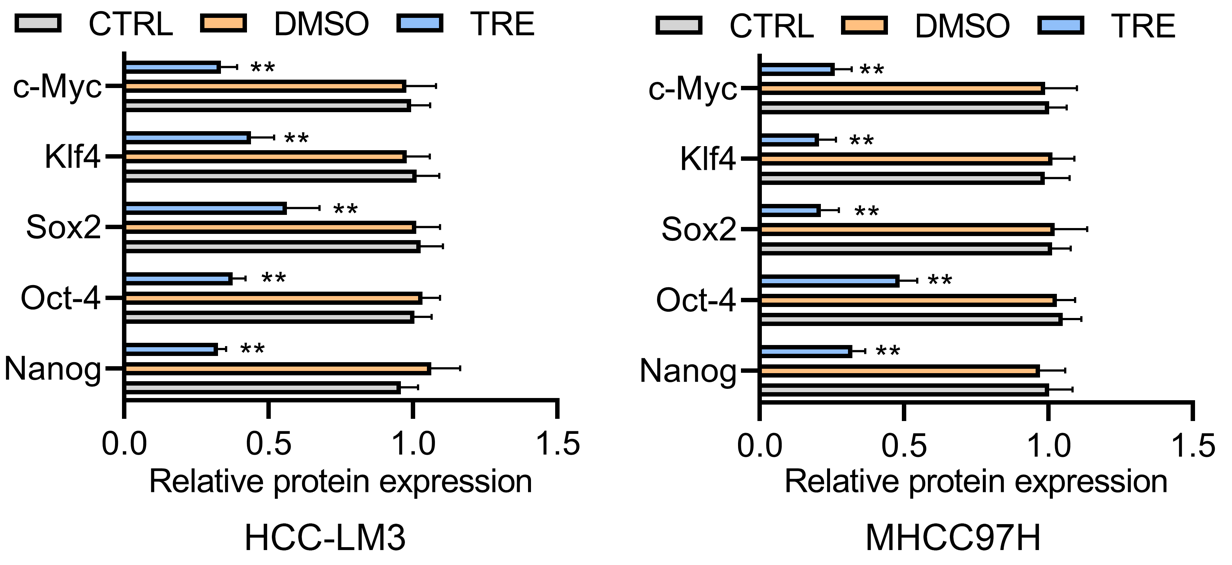


**Figure S1. Quantification of the Western blot data shown in Figure 2D.** Relative protein levels of Nanog, Oct-4, Sox2, Klf4, and c-Myc in HCC-LM3 and MHCC97H cells from the CTRL, DMSO, and TRE groups were determined by densitometric analysis and normalized to GAPDH. Mean ± SD, n = 3 independent experiments. ***P* < 0.01.


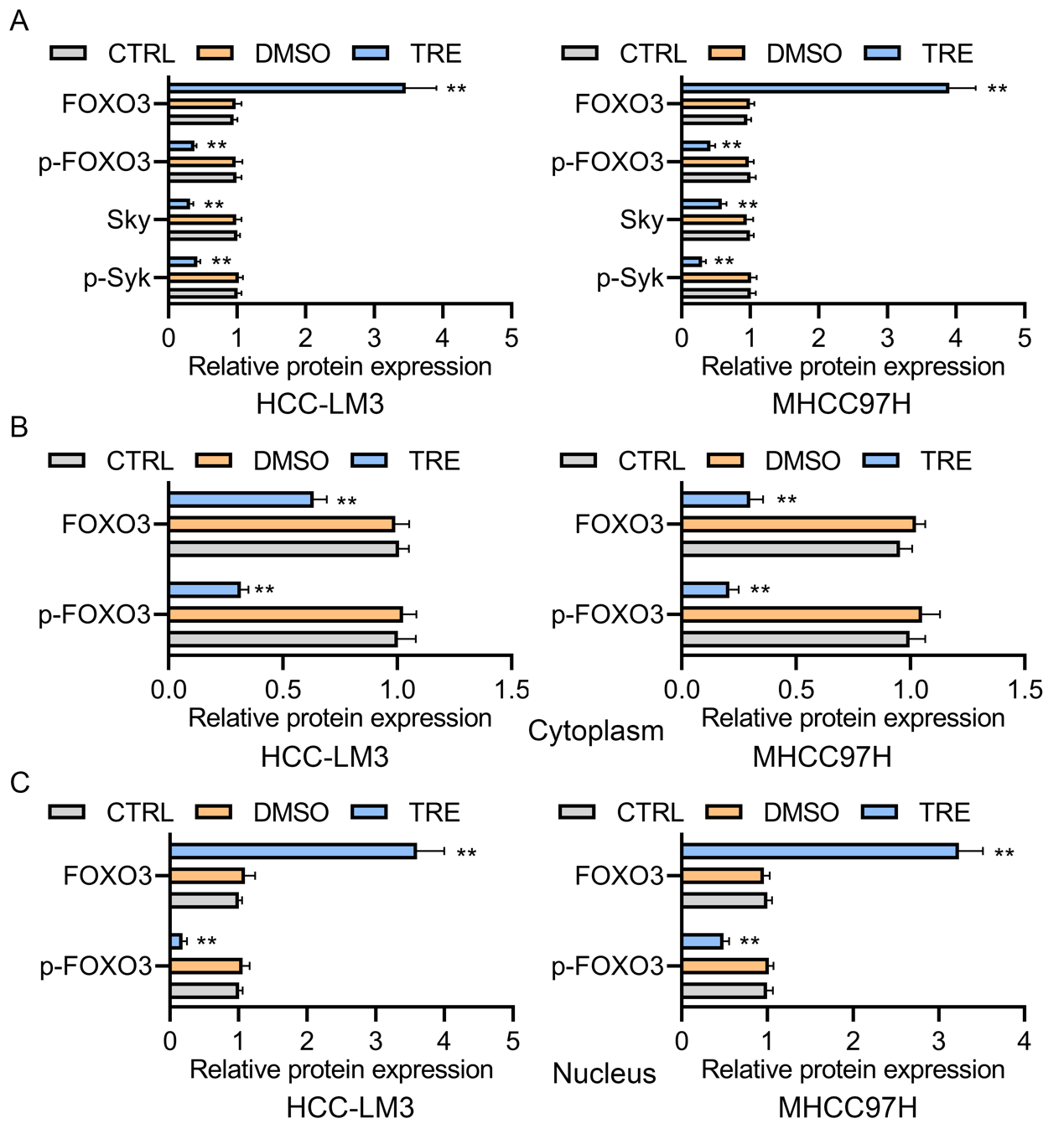


**Figure S2. Quantitative analysis of the Western blot data shown in Figure 3F–G.** (**A**) Relative levels of p-Syk, Syk, p-FOXO3, and FOXO3 in HCC-LM3 and MHCC97H cells from the CTRL, DMSO, and TRE groups. (**B–C**) Relative levels of p-FOXO3 and FOXO3 in the cytoplasmic (**B**) and nuclear (**C**) fractions of HCC-LM3 and MHCC97H cells from the CTRL, DMSO, and TRE groups. Data are mean ± SD, n = 3 independent experiments. ***P* < 0.01.


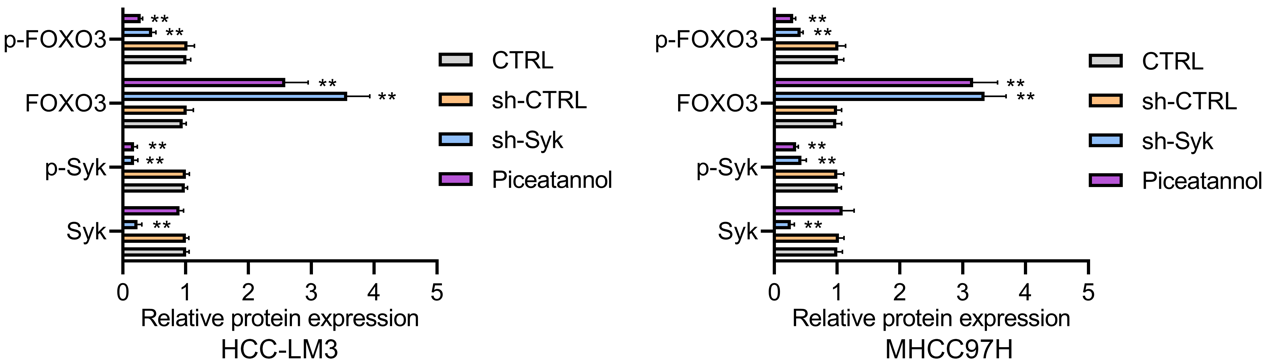


**Figure S3.** **Densitometric analysis of the Western blot data shown in Figure 4A.** Relative protein levels of Syk, p-Syk, FOXO3, and p-FOXO3 in HCC-LM3 and MHCC97H cells from the CTRL, sh-CTRL, sh-Syk, and piceatannol groups. Mean ± SD, n = 3 independent experiments. ***P* < 0.01.


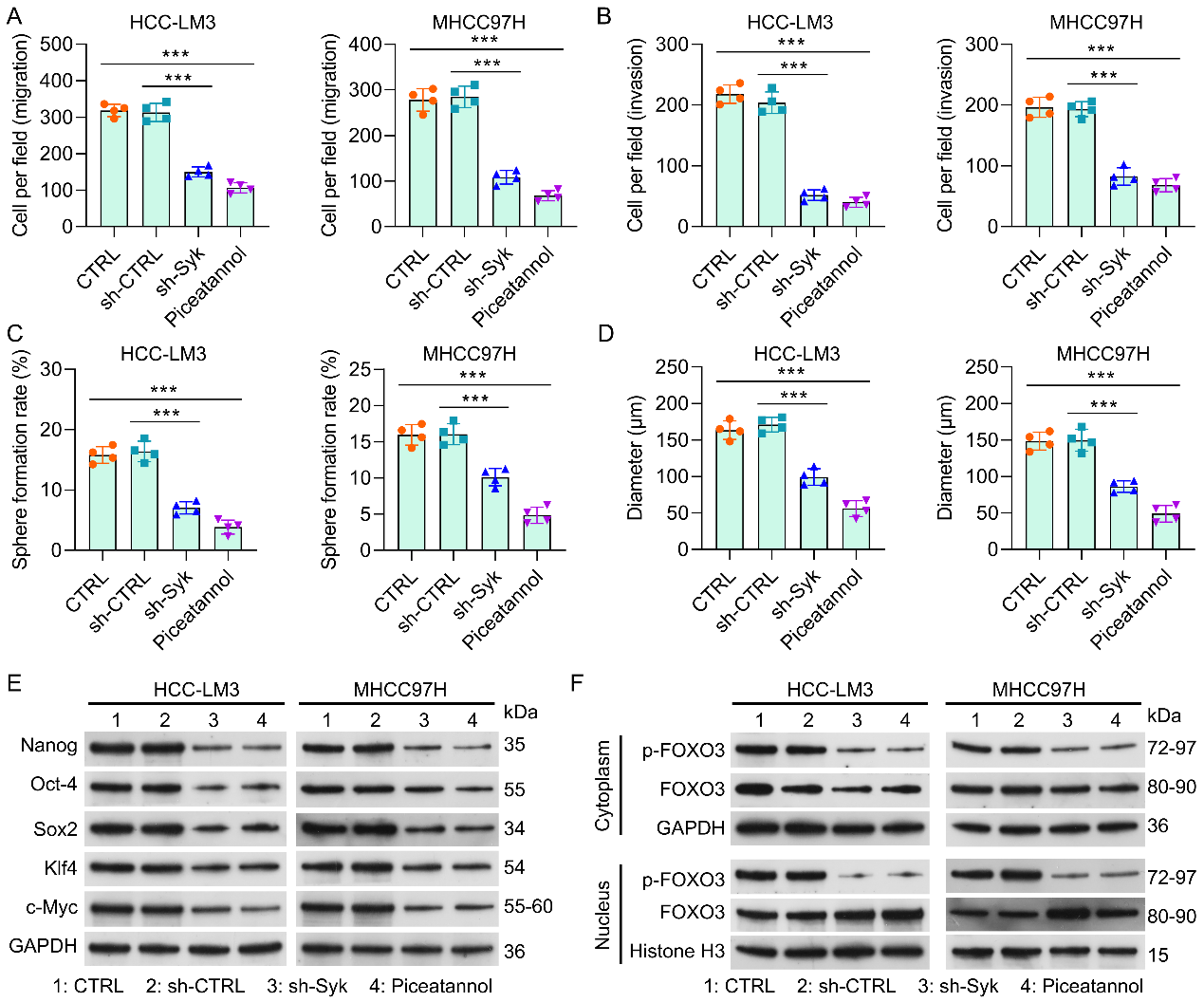


**Figure S4. Effects of Syk inhibition on malignant phenotypes and stemness-associated markers in HCC cells. (A–B)** Quantitative analysis of Transwell migration and invasion assays across four experimental groups (CTRL, sh‑CTRL, sh‑Syk, and piceatannol) (n = 3). (**C–D**) Sphere formation assay results showing sphere number and diameter in the same four cohorts of HCC‑LM3 and MHCC‑97H cells. (**E**) Western blot analysis of stemness markers Sox2, Nanog, Klf4, c‑Myc, and OTC4 in the four cohorts. (**F**) Cytoplasmic and nuclear fractions were isolated for Western blotting of p‑FOXO3, with GAPDH and Histone H3 serving as cytoplasmic and nuclear markers, respectively. Data are mean ± SD, n = 3 independent experiments. ****P* < 0.001.

**
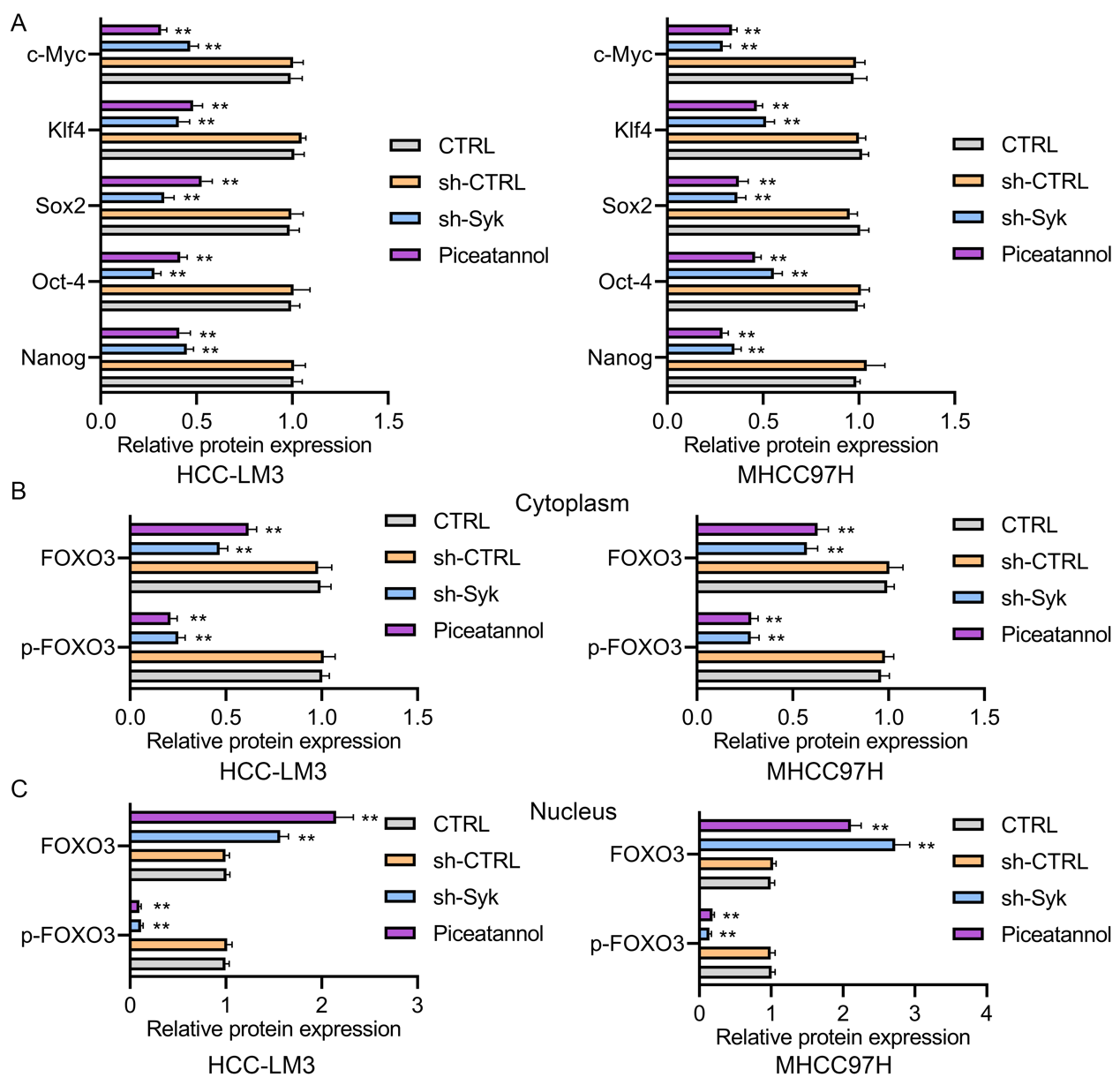
**

**Figure S5. Quantification of the Western blot data in Figure S4E–F.** (**A**) Relative levels of Nanog, Oct-4, Sox2, Klf4, and c-Myc in HCC-LM3 and MHCC97H cells from the CTRL, sh-CTRL, sh-Syk, and piceatannol groups. (**B–C**) Relative levels of p-FOXO3 and FOXO3 in the cytoplasmic (**B**) and nuclear (**C**) fractions of HCC-LM3 and MHCC97H cells from the indicated groups. Data are mean ± SD, n = 3 independent experiments. ***P* < 0.01.

**
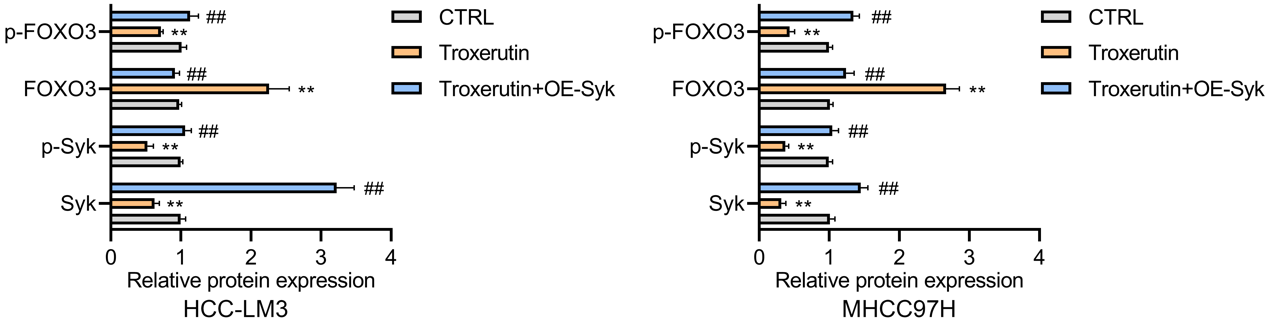
**

**Figure S6. Quantification of the Western blot data shown in Figure 5A.** Relative protein levels of Syk, p-Syk, FOXO3, and p-FOXO3 were measured in HCC-LM3 and MHCC97H cells from the CTRL, Troxerutin, and Troxerutin+OE-Syk groups. Data are mean ± SD, n = 3 independent experiments. ***P* < 0.01 vs. CTRL group; ##*P* < 0.01 vs. Troxerutin group.


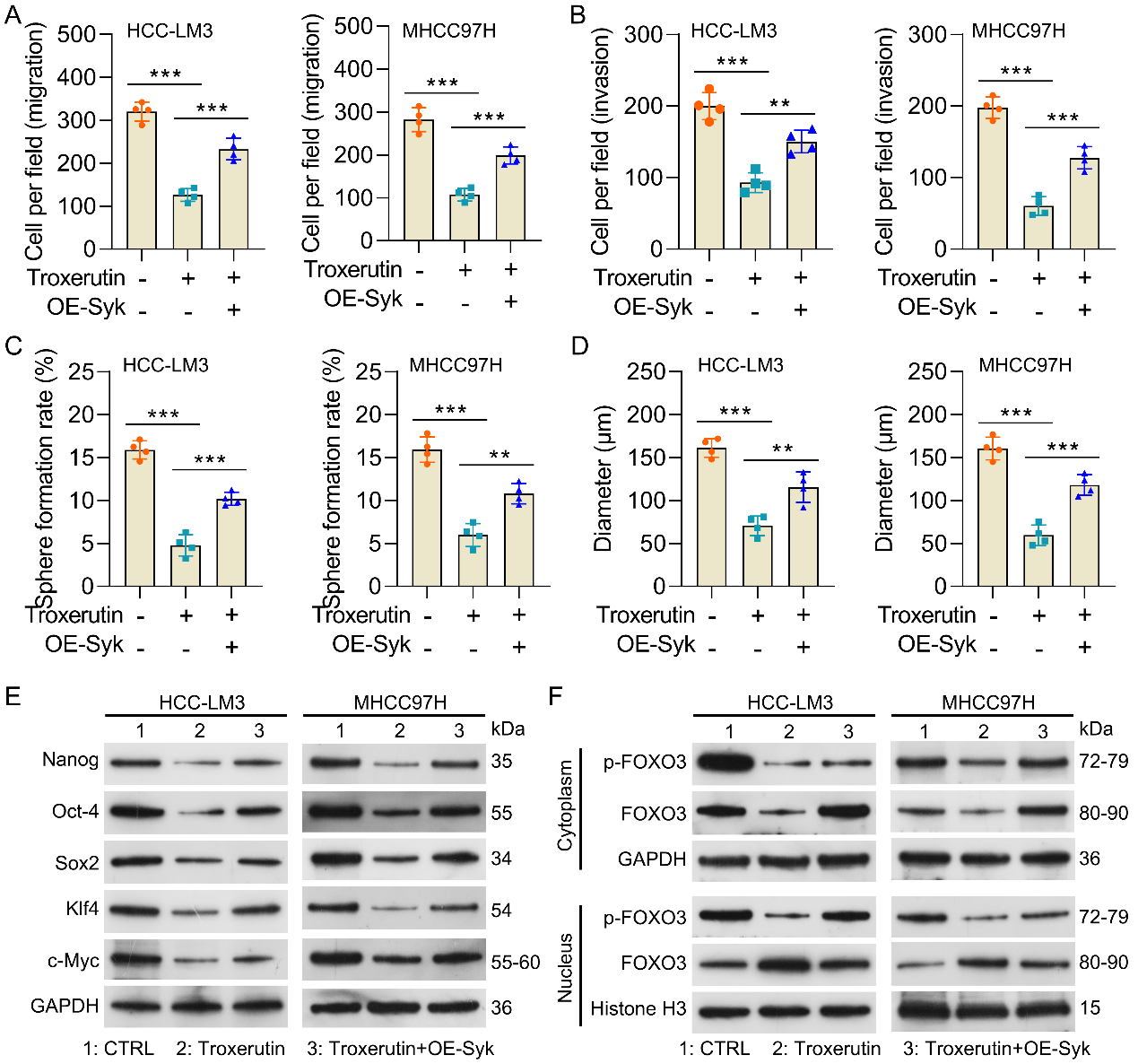


**Figure S7. Effects of Syk overexpression on malignant phenotypes and stemness-associated markers in troxerutin-treated HCC cells. (A–B)** Quantitative analysis of Transwell migration and invasion assays across three experimental groups (CTRL, troxerutin, and troxerutin + OE‑Syk) (n = 3). (**C–D**) Sphere formation assay results showing sphere number and diameter in the same three cohorts of HCC‑LM3 and MHCC‑97H cells. **(E)** Stemness markers Sox2, Nanog, Klf4, c-Myc, and OTC4 were measured by Western blotting in the four cohorts (CTRL, sh-CTRL, sh-Syk, and piceatannol), as detected by Western blot. **(F)** After cytoplasmic and nuclear proteins were isolated and put through Western blot, p-FOXO3 and FOXO3 were found in the cytoplasmic and nuclear fractions, respectively. The cytoplasm and nucleus were identified by measuring GAPDH and Histone H3, respectively. Mean ± SD, n = 3 independent experiments. ***P* < 0.01; ****P* < 0.001.


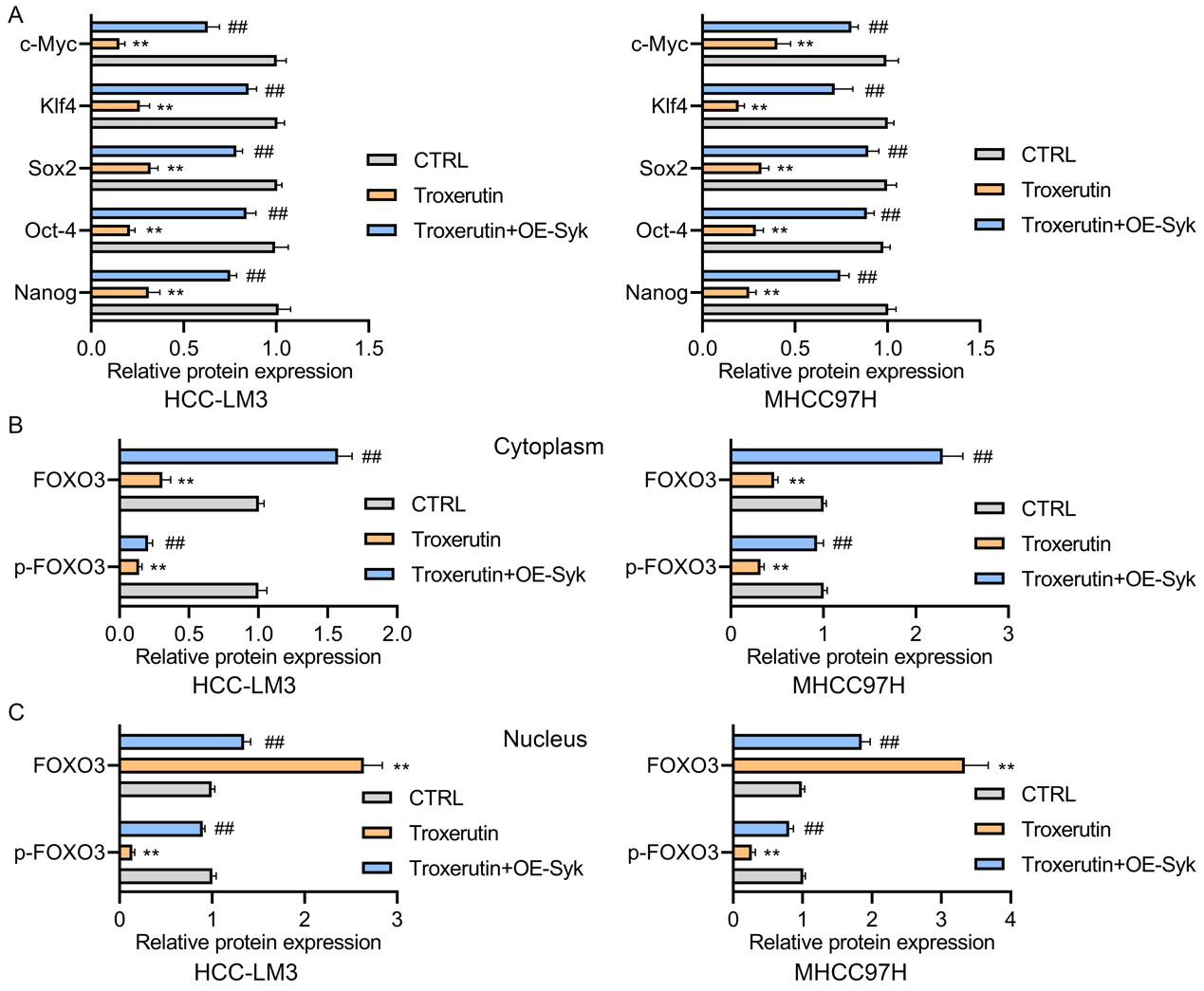


**Figure S8.** **Figure S8. Quantification of the Western blot data in Figure S7E–F.** HCC-LM3 and MHCC97H cells were analyzed in the CTRL, troxerutin, and troxerutin+OE-Syk groups. (**A**) Relative protein levels of Nanog, Oct-4, Sox2, Klf4, and c-Myc. (**B–C**) Relative levels of p-FOXO3 and FOXO3 in the cytoplasmic (**B**) and nuclear (**C**) fractions. Data are mean ± SD, n = 3 independent experiments. ***P* < 0.01 vs. CTRL group; ##*P* < 0.01 vs. Troxerutin group.


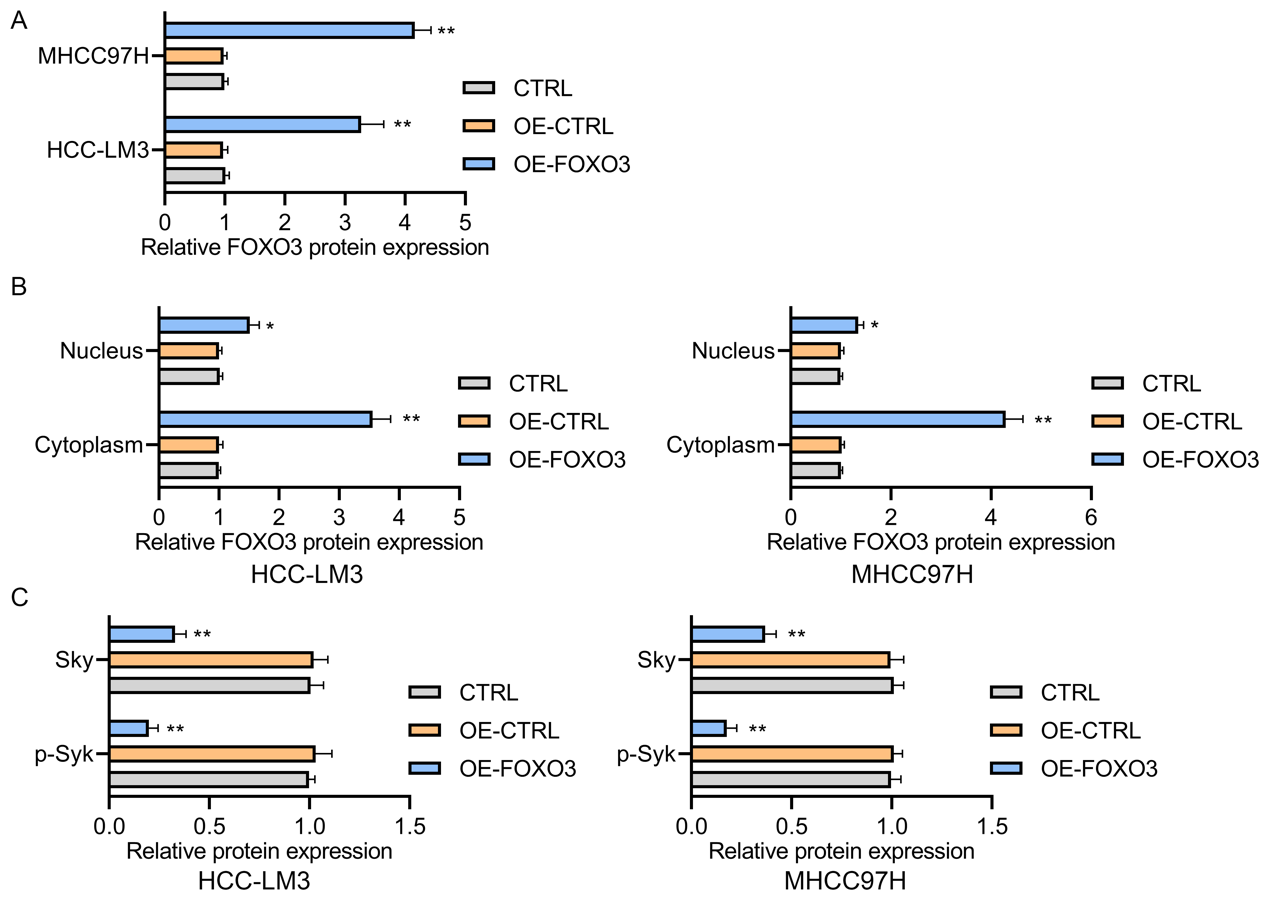


**Figure S9. Quantification of the Western blot data in Figure 6E–G.** HCC-LM3 and MHCC97H cells were analyzed in the CTRL, OE-CTRL, and OE-FOXO3 groups. (**A**) Relative FOXO3 protein levels. (**B**) Relative FOXO3 protein levels in the cytoplasmic and nuclear fractions. (**C**) Relative Syk and p-Syk protein levels. Data are mean ± SD, n = 3 independent experiments. **P* < 0.05; ***P* < 0.01.


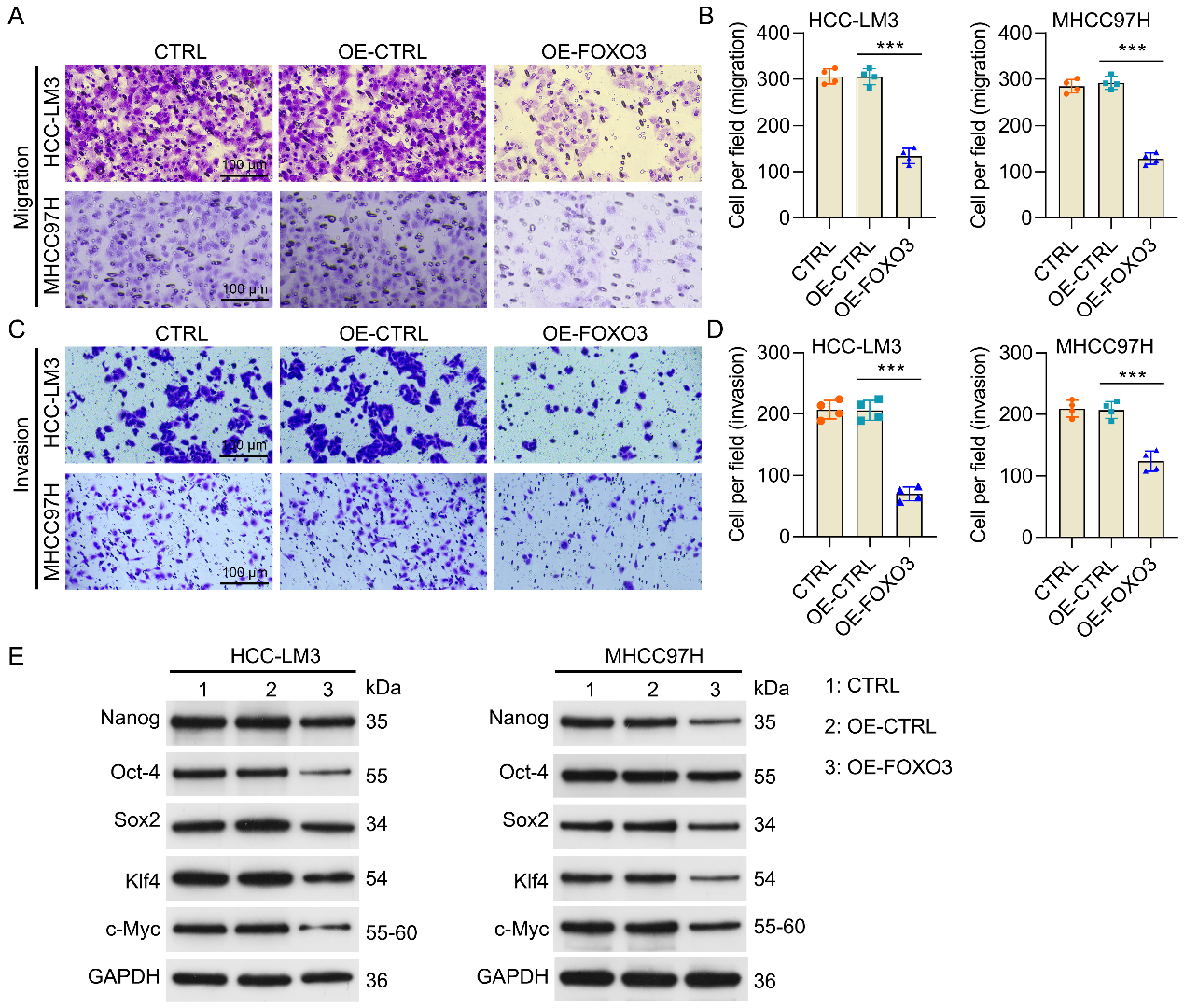


**Figure S10.** **Effects of FOXO3 overexpression on malignant phenotypes and stemness-related proteins in HCC cells. (A–D)** Migration and invasion capacities of HCC cells after transfection with FOXO3 overexpression plasmid, as evaluated by Transwell assays. (**E**) Western blot analysis of stemness markers Sox2, Nanog, Klf4, c‑Myc, and OTC4 in control and FOXO3‑overexpressing cells. Data are presented as mean ± SEM from at least three independent replicates. Data are mean ± SD, n = 3 independent experiments. ****P*  <  0.001.


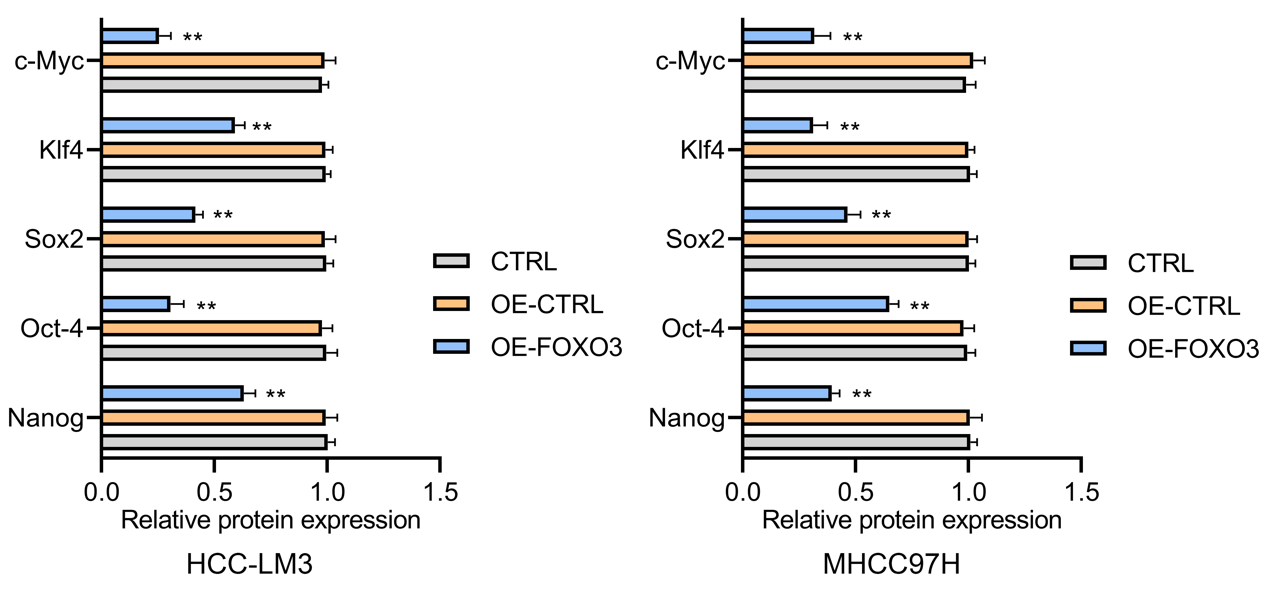


**Figure S11.** **Quantification of the Western blot data in Figure S10E.** Relative levels of Nanog, Oct-4, Sox2, Klf4, and c-Myc in HCC-LM3 and MHCC97H cells from the CTRL, OE-CTRL, and OE-FOXO3 groups. Data are mean ± SD, n = 3 independent experiments. ***P* < 0.01.


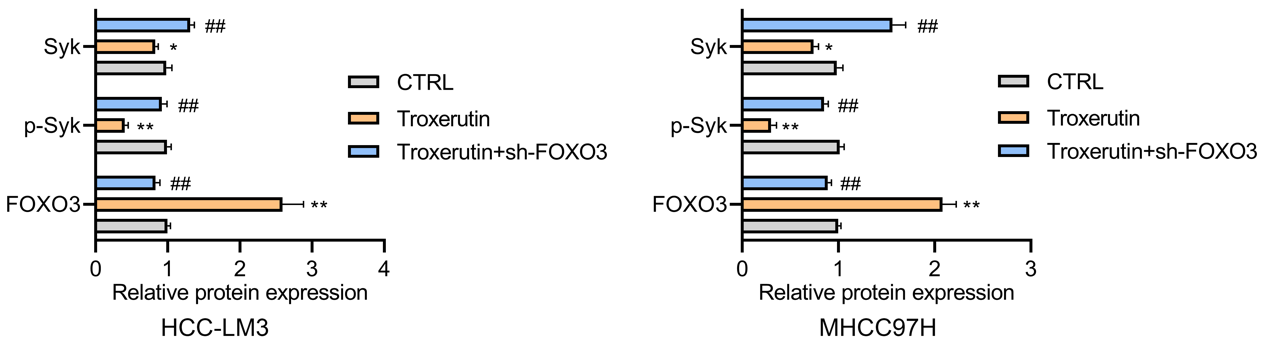


**Figure S12. Quantification of the immunoblot data in Figure 8A.** Relative levels of FOXO3, Syk, and p-Syk in HCC-LM3 and MHCC97H cells from the CTRL, troxerutin, and troxerutin+sh-FOXO3 groups. Data are mean ± SD, n = 3 independent experiments. **P* < 0.05, ***P* < 0.01 vs. CTRL group; ##*P* < 0.01 vs. Troxerutin group.


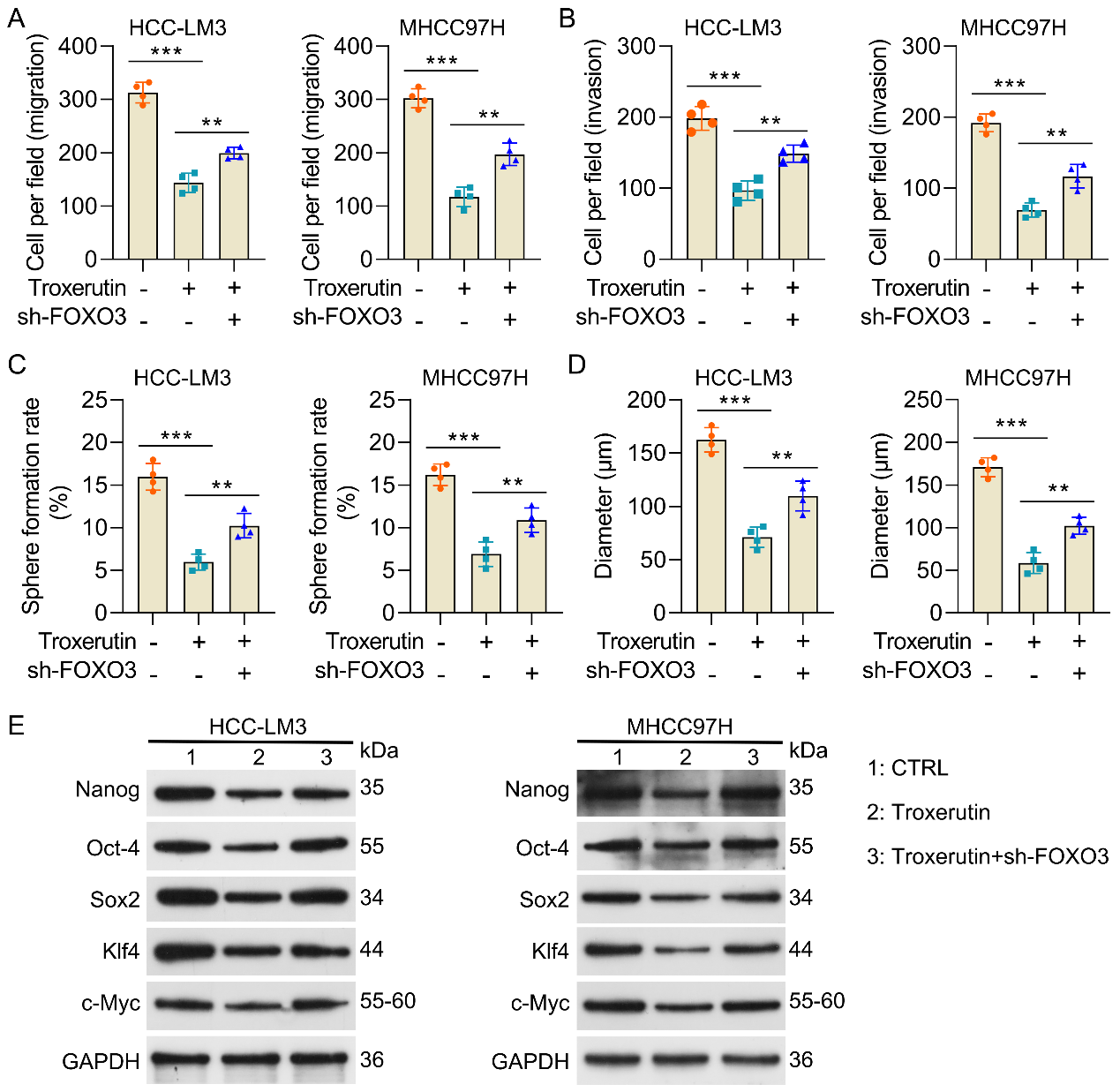


**Figure S13.** **Effects of FOXO3 knockdown on malignant phenotypes and stemness-related proteins in troxerutin-treated HCC cells. (A–D)** Transwell assays were used to evaluate the migration and invasion potential of HCC cells treated with troxerutin and transfected with sh-FOXO3 (**E**). Western blot analysis of stemness markers Sox2, Nanog, Klf4, c‑Myc, and OTC4 in the indicated groups. Data are mean ± SD, n = 3 independent experiments. ****P* < 0.001.


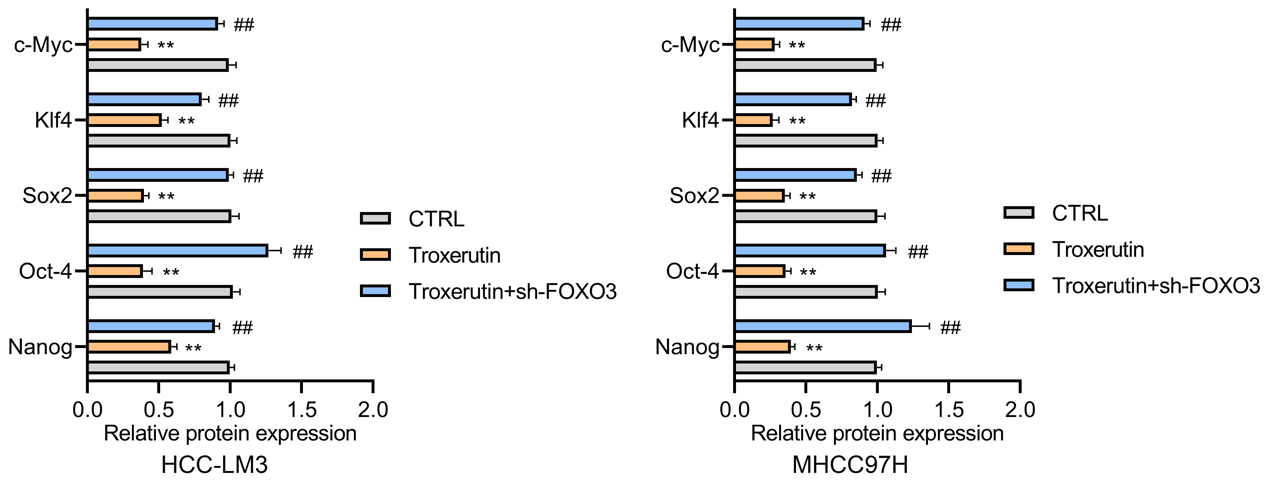


**Figure S14.** **Quantification of the immunoblot data in Figure S13E.** Relative levels of Nanog, Oct-4, Sox2, Klf4, and c-Myc in HCC-LM3 and MHCC97H cells from the CTRL, troxerutin, and troxerutin+sh-FOXO3 groups. Data are mean ± SD, n = 3 independent experiments. ***P* < 0.01 vs. CTRL group; ##*P* < 0.01 vs. Troxerutin group.


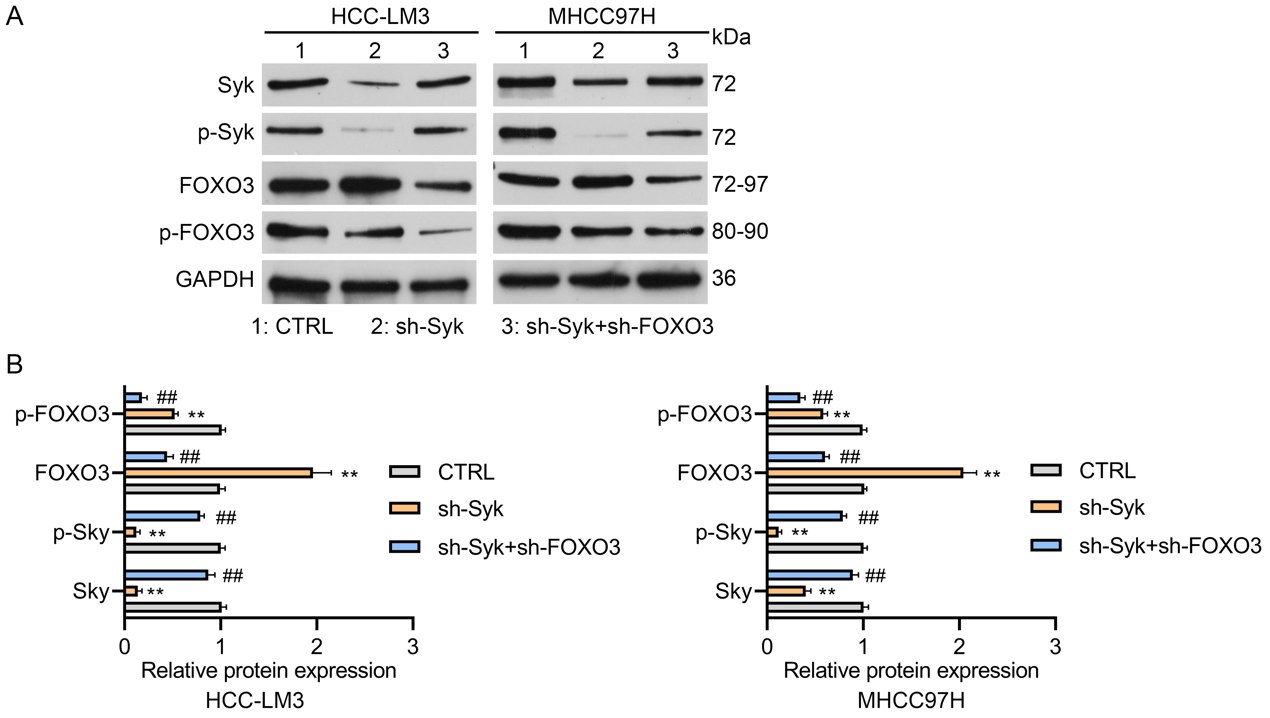


**Figure S15. Western blotting analysis and quantification of Syk/FOXO3 pathway proteins in HCC cells.** (**A**) Western blot analysis of Syk, p-Syk, FOXO3, and p-FOXO3 in HCC-LM3 and MHCC97H cells from the CTRL, sh-Syk, and sh-Syk+sh-FOXO3 groups. (**B**) Densitometric analysis of the immunoblot data. Mean ± SD, n = 3 independent experiments. ***P* < 0.01 vs. CTRL group; ##*P* < 0.01 vs. sh-Syk group.


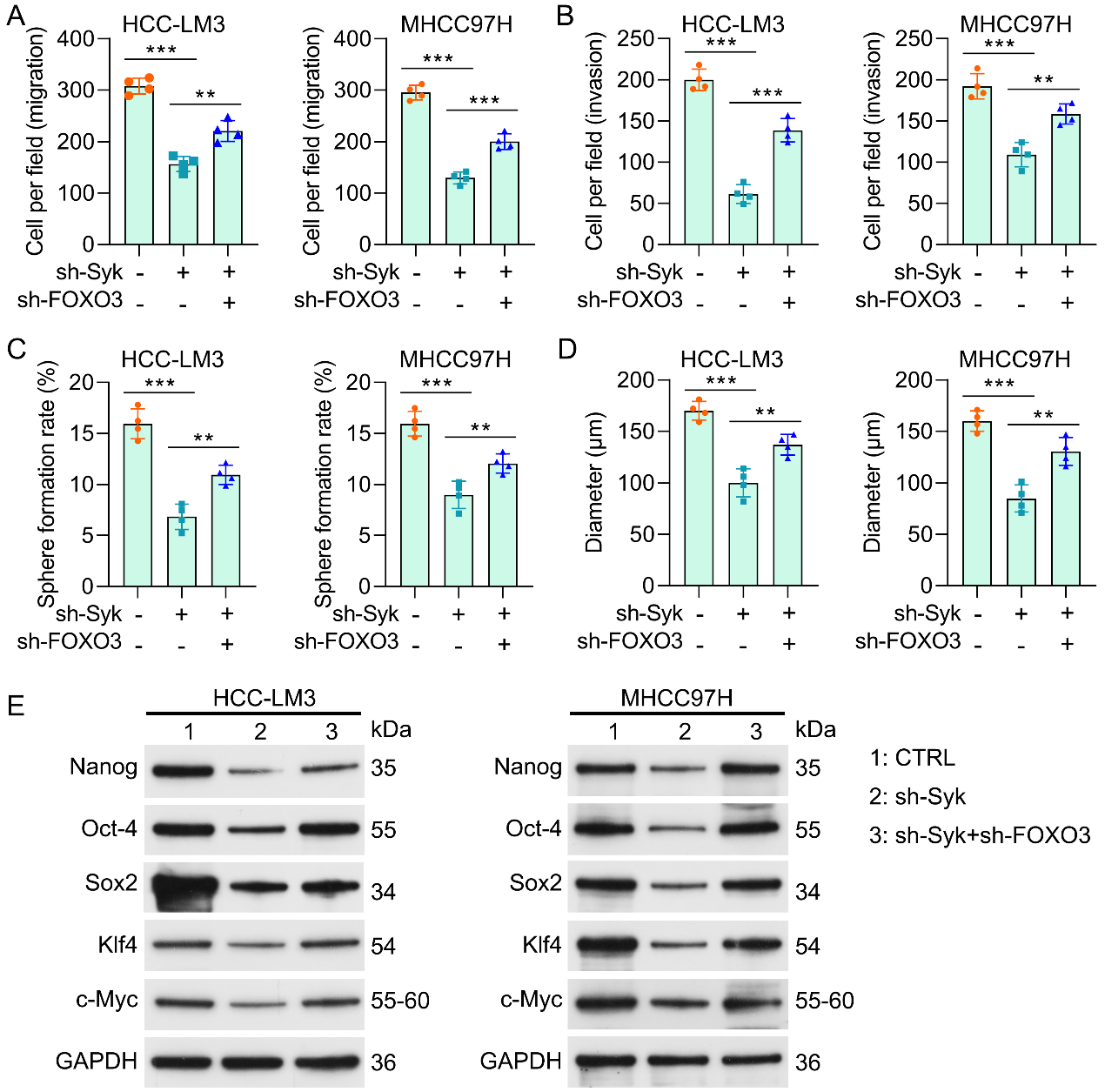


**Figure S16. Effects of FOXO3 knockdown on malignant phenotypes and stemness-related proteins in Syk-silenced HCC cells. (A–B)** Transwell assays were used to evaluate the migration and invasion capabilities of HCC cells with Syk downregulation after sh-FOXO3 transfection. (**C–D**) Quantification and diameter measurements of spheres formed in the in-vitro sphere formation assay for the indicated groups. (**E**) Western blot analysis of stemness markers Sox2, Nanog, Klf4, c‑Myc, and OTC4. Data are presented as mean ± SEM from at least three independent experiments. Data are mean ± SD, n = 3 independent experiments. ***P* <  0.01, ****P* <  0.001.


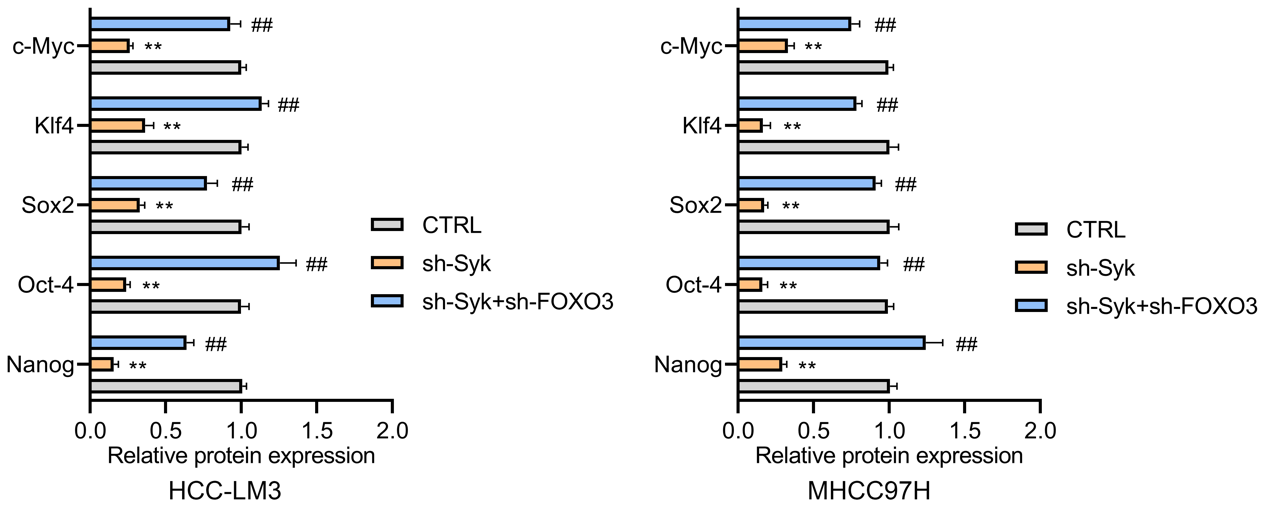


**Figure S17. Figure S17. Quantification of the immunoblot data in Figure S16E.** Relative levels of Nanog, Oct-4, Sox2, Klf4, and c-Myc in HCC-LM3 and MHCC97H cells from the CTRL, sh-Syk, and sh-Syk+sh-FOXO3 groups. Data are mean ± SD, n = 3 independent experiments. ***P* < 0.01 vs. CTRL group; ##*P* < 0.01 vs. sh-Syk group.


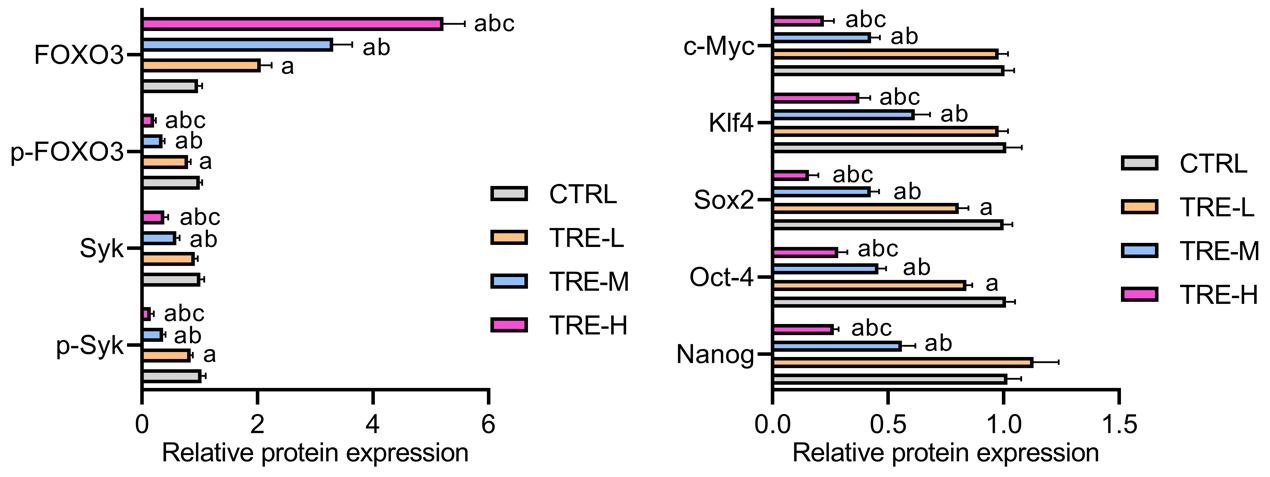


**Figure S18. Quantification of the immunoblot data in Figure 10H–I.** Relative levels of Syk, p-Syk, FOXO3, and p-FOXO3, as well as Nanog, Oct-4, Sox2, Klf4, and c-Myc, in tumor tissues from the CTRL, TRE-L, TRE-M, and TRE-H groups. Mean ± SD, n = 3 independent experiments. Compared with CTRL, a *P* < 0.05, compared with TRE-L, b *P* < 0.05, compared with TRE-M, c *P* < 0.05.
